# Powdery mildew fungal effector candidates share N-terminal Y/F/WxC-motif

**DOI:** 10.1186/1471-2164-11-317

**Published:** 2010-05-20

**Authors:** Dale Godfrey, Henrik Böhlenius, Carsten Pedersen, Ziguo Zhang, Jeppe Emmersen, Hans Thordal-Christensen

**Affiliations:** 1 Plant and Soil Science Laboratory, Department of Agricultural and Ecology, Faculty of Life Sciences, University of Copenhagen, Denmark; 2 Department of Health Science and Technology, Aalborg University, Denmark

## Abstract

**Background:**

Powdery mildew and rust fungi are widespread, serious pathogens that depend on developing haustoria in the living plant cells. Haustoria are separated from the host cytoplasm by a plant cell-derived extrahaustorial membrane. They secrete effector proteins, some of which are subsequently transferred across this membrane to the plant cell to suppress defense.

**Results:**

In a cDNA library from barley epidermis containing powdery mildew haustoria, two-thirds of the sequenced ESTs were fungal and represented ~3,000 genes. Many of the most highly expressed genes encoded small proteins with N-terminal signal peptides. While these proteins are novel and poorly related, they do share a three-amino acid motif, which we named "Y/F/WxC", in the N-terminal of the mature proteins. The first amino acid of this motif is aromatic: tyrosine, phenylalanine or tryptophan, and the last is always cysteine. In total, we identified 107 such proteins, for which the ESTs represent 19% of the fungal clones in our library, suggesting fundamental roles in haustoria function. While overall sequence similarity between the powdery mildew Y/F/WxC-proteins is low, they do have a highly similar exon-intron structure, suggesting they have a common origin. Interestingly, searches of public fungal genome and EST databases revealed that haustoria-producing rust fungi also encode large numbers of novel, short proteins with signal peptides and the Y/F/WxC-motif. No significant numbers of such proteins were identified from genome and EST sequences from either fungi which do not produce haustoria or from haustoria-producing Oomycetes.

**Conclusion:**

In total, we identified 107, 178 and 57 such Y/F/WxC-proteins from the barley powdery mildew, the wheat stem rust and the wheat leaf rust fungi, respectively. All together, our findings suggest the Y/F/WxC-proteins to be a new class of effectors from haustoria-producing pathogenic fungi.

## Background

Biotrophic plant fungal pathogens cause dramatic yield losses in many crop species worldwide. Graminaceous powdery mildew and rust diseases are particularly serious because of the extensive cultivation of their hosts, wheat, barley and maize. However, sorghum, rye, oat and a number of forage grasses also commonly suffer from attack by these pathogens. The importance of powdery mildew has motivated Pietro Spanu, Imperial College, UK to release preliminary genome sequence data of the barley powdery mildew fungus (*Blumeria graminis *f.sp. *hordei*, *Bgh*) as model organism for this unique group of pathogens at http://www.ncbi.nlm.nih.gov. In recent years, major outbreaks of wheat stem rust epidemics, caused by a new aggressive fungal isolate (Ug99), have led to very significant losses in Africa [1]. This has spurred genome sequencing of the wheat stem rust fungus (*Puccinia graminis *f.sp. *tritici*, *Pgt*). See http://www.broad.mit.edu/annotation/genome/puccinia_graminis.3/home.html.

Biotrophic plant pathogens live in intimate contact with their hosts and are dependent on an ability to transfer effector proteins into cells of the host. We define effectors as proteins and other compounds that enhance disease development by targeting host processes, but are otherwise redundant to basal growth processes in the pathogen. A primary role of effectors is to inhibit host defense mechanisms [2-4]. The filamentous powdery mildew and rust fungi, as well as many Oomycete pathogens, all develop haustoria within the lumen of the host cell, where they serve to take up nutrients. In parallel with the growth of the haustorium, the host cell generates a membrane of unknown origin, which surrounds the haustorium. Many bacterial pathogens use the "type three secretion system" (T3SS) to inject effectors into plant cells [3]. However, it remains unsolved how haustoria-forming fungal pathogens transfer effectors into the host cell, where some of these proteins confer their function. It is known that Oomycete effectors are dependent on N-terminal signal peptides for secretion from the haustoria, via the default secretory pathway. Furthermore, these effectors carry an amino acid double motif (RxLR-dEER) required for them to cross the plant-derived extrahaustorial membrane (EHM). This motif is located a few amino acids downstream of the signal peptide cleavage sites [5,6]. The RxLR-dEER double motif has been used to screen genome sequences and allowed identification of approximately 700 *Phytophthora *[7,8] and 150 *Hyaloperonospora *effector candidates [9]. Based on bacterial genome sequences and the T3SS target signal, inventories have been obtained each with a few dozen bacterial effector candidates [10]. Effector candidates generally are small proteins in their mature form, and they rarely have homologues in more remotely related microbial species [7,8,11].

Very few effectors and effector candidates have been identified from haustoria-forming fungal pathogens. Two effectors have been identified from the barley powdery mildew fungus, *Bgh *[2], and a number of effectors and effector candidates have been identified from rust fungi [12,13]. *Bgh *and other powdery mildew fungi are highly suitable for haustoria EST sequencing, since they only attack the above ground plant epidermal cell layer, which allows specific sampling of tissue highly enriched with haustoria. This led us to conduct EST sequencing in an attempt to identify new *Bgh *effectors. In the present study, we identified more than 100 effector candidate proteins, all sharing a "Y/F/WxC"-motif in the N-terminal, downstream of signal peptides. Subsequent studies of other pathogen genomes revealed that the wheat stem rust and wheat leaf rust fungi also have large sets of genes encoding proteins of similar structure as the *Bgh *effector candidates.

## Results

### Highly expressed *Bgh *haustorial genes encode novel effector candidates with signal peptides

**Figure 1:**
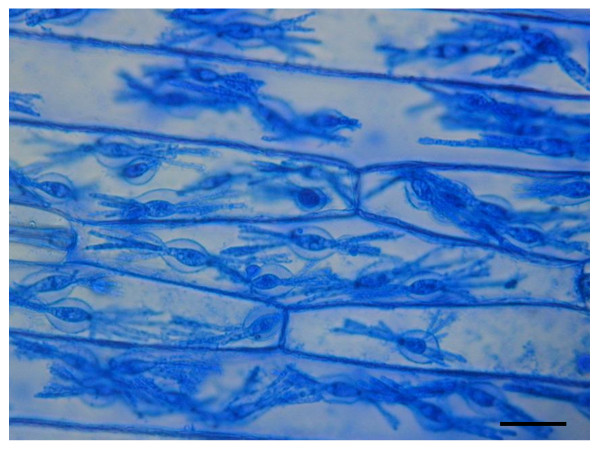
**Barley epidermal strips containing *Bgh *haustoria used to generate cDNA library**. At 7 days post inoculation the surface fungal material was removed prior to sampling and the strip was stained with Coomassie Brilliant Blue to visualize haustoria. mRNA was extracted from comparable tissue and used to generate the described cDNA library. Size bar, 20 μm.

*Bgh *establishes the first haustorium in the barley leaf epidermal cells from approximately 15 hours post inoculation (hpi). Conidia that fail to generate haustoria die. After day 1, colony formation starts, and during the first week, hyphal growth and formation of new conidia will occur unhindered. Secondary haustoria are established from day 2-3, and by the late stage of infection at day 6-7, very large numbers of haustoria are present in the host epidermal cells, as seen in Figure 1. Aiming to obtain plant tissue with the highest possible content of fungal haustoria, we chose to collect epidermal material at this late stage of infection (7 dpi). Surface fungal hyphae and spores were removed prior to tissue sampling. This material was used to generate a cDNA library, of which 9,928 EST reads were made. Cluster analysis was followed by BLAST searches against public databases to predict whether the sequences were of fungal or plant origin. This revealed that approximately two-thirds of the ESTs, representing fungal transcripts expressed in the *Bgh *haustorium, clustered into approximately 3,200 unigenes. Approximately 90% of these were present in the preliminary *Bgh *genome sequence data at http://www.ncbi.nlm.nih.gov. The cluster analysis showed that the most frequently occurring fungal EST sequence appeared 110 times. This EST contig encodes a 109-amino acid protein, *Bgh*Efc1, with a predicted N-terminal signal peptide. The 88-amino acid mature protein has no homologues outside *B. graminis*. We then listed the fungal contigs according to their number of EST sequences. Interestingly, at the top of the list, where the contigs were assembled from 110 to 9 ESTs, approximately 40% of them encoded small, novel proteins with predicted N-terminal signal peptides. Using this approach, we initially identified 35 contigs with these characteristics, encoding proteins of 106 to 161 amino acids (Additional file 1: Table S1). Expression analysis of 23 transcripts from this group demonstrated that all were expressed predominantly in haustoria (see below). The haustoria expression and the predicted signal peptide allow us to term these 35 proteins that are unique to *B. graminis*, "effector candidates" or *Bgh*Efc's.

### Effector candidates share a Y/F/WxC-motif

Inspired by the RxLR-dEER motif required for EHM transfer of Oomycete effectors, we searched for a motif downstream of the predicted signal peptide in the 35 *Bgh *effector candidate proteins. An alignment of these mature proteins revealed a "Y/F/WxC"-amino acid motif within 17 amino acids of the predicted signal peptide cleavage sites (Additional file 2: Figure S1). In the majority of the proteins (23), the first amino acid of the motif is tyrosine (Y), to a lesser extent (10) it is phenylalanine (F) and in a few cases (2) it is tryptophan (W). These three amino acids constitute the group of uncharged aromatic amino acids, which share considerable structural similarity.

**Figure 2:**
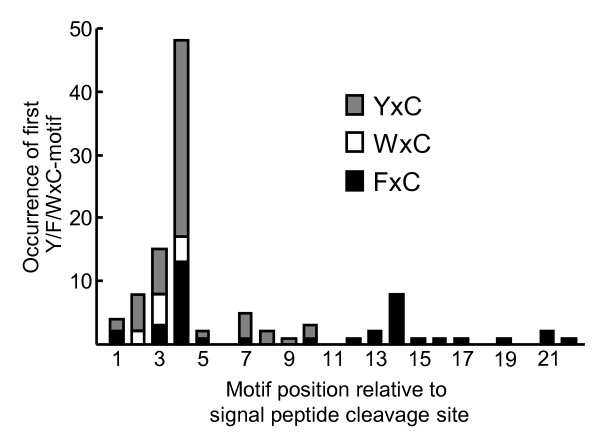
**The Y/F/WxC-motif in *Bgh *effector candidates predominantly starts at amino acid position 4**. The cumulative number of the YxC, WxC and FxC versions of the Y/F/WxC-motif plotted according to the position of the first amino acid of the motif, relative to the signal peptide cleavage site. Only the first Y/F/WxC-motif in each protein is included.

We subsequently examined all fungal proteins that could be identified from the EST library and found in total 107 EST clusters and singletons that predominantly encode short (48-228 amino acids), *B. graminis*-specific proteins that have a predicted N-terminal signal peptide, and importantly have the Y/F/WxC-motif within the first 45 amino acids of the N-terminal methionine. In all cases, the Y/F/WxC-motif is located within 24 amino acids downstream from the predicted signal peptide cleavage site. Of these 107 EST clusters and singletons, 42 were not full-length. For 40 of those, we were able to identify the complete coding sequences by help of the partial *Bgh *genome sequence at http://www.ncbi.nlm.nih.gov. Additional file 1: Table S1 lists all 107 *Bgh *proteins identified using this approach. It appears that the Y, F and W at the first position occurred in 56, 40 and 11 of the proteins, respectively, that the central amino acid of the Y/F/WxC-motif never is cysteine (C), and that the last amino acid always is C (Additional file 1: Table S1). Figure 2 illustrates the distribution and nature of the Y/F/WxC-motif along the N-terminus of the mature effector candidates. This shows that the motif starts in position 4 in 49 of these 107 proteins, suggesting this to be the optimal position of the motif relative to a protein N-terminus. In most cases (93 of 107 sequences) the *Bgh *effector candidates only have one Y/F/WxC-motif. However, two and three copies of the motif are present in 12 and two of the sequences, respectively (Additional file 1: Table S1). The total number of ESTs encoding Y/F/WxC-effector candidates sums up to 1,223. This is as much as 19% of all haustorial ESTs in the library, emphasizing the importance of these proteins for this fungal structure. The 110 ESTs of *BghEfc1 *alone constitute 1.7%.

Only three of the proteins encoded by these highly expressed transcripts, *BghEfc8*, *BghEfc13 *and *BghEfc54*, were found among 204 fungal proteins identified in the haustorial proteome ([14], in which these three proteins were referred to as CL14C1, CL22C2 and CL134C1, respectively). This suggests that even though many of the 107 Y/F/WxC-genes are highly expressed, the proteins in general are secreted from the haustoria (see Additional file 1: Table S1). Furthermore, five short, unknown proteins with signal peptide, found in a proteome study of haustoria-containing epidermal tissue [15], are represented within the 107 Y/F/WxC-effector candidates (Additional file 1: Table S1). In this study a total of 47 *Bgh *proteins were identified [15]. The five proteins were not identified within the haustoria proteome [14], suggesting they are secreted.

### The *Bgh *Y/F/WxC-effector candidates are highly diverse, yet they do share a conserved gene structure

**Figure 3:**
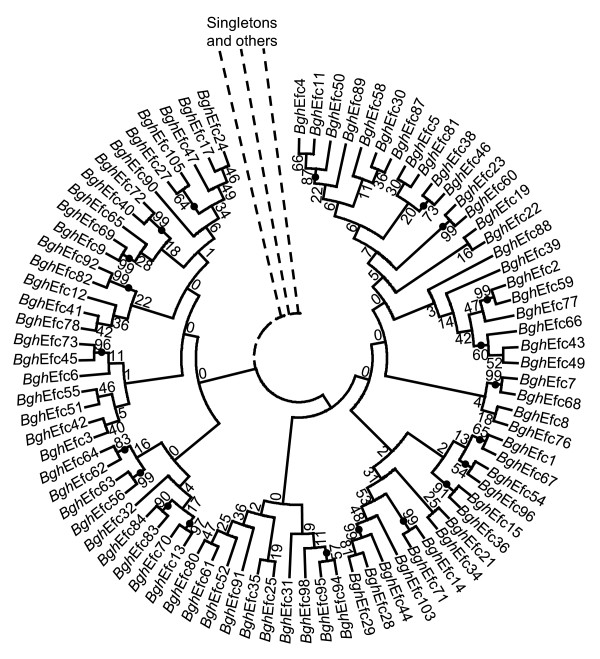
**Phylogenetic analysis supports the existence of small families of *Bgh *effector candidates**. The unrooted phylogenetic tree is based on NJ analysis. Confidence of groupings was estimated by using 1,000 bootstrap replicates and the percentage of replicate trees supporting each branch are shown next to the branching point. Clades supported by bootstrap value larger than 50 are indicated by black dots, unless a lower partitioning point with a lower bootstrap value occurs.

BLAST searches against the NCBI nr/nt database could not identify homologues outside of *B. graminis*. When compared to each other, their relationship is also poor. In a multiple alignment of the 107 mature proteins, the Y/F/WxC-motif aligns in 104 of the sequences (Additional file 3: Figure S2). No other sequence structure reaches this level of conservation. However, based on shared sequence identity, 83 of the 105 full-length *Bgh *effector candidates group into 17 clusters. The clusters, ranging in size from two to 17 proteins, are based on the criteria that all members have at least one BLAST hit (*E*-value ≤1e-5) to another member of the cluster (Additional file 1: Table S1). The remaining 22 are singletons which do not have a significant BLAST hit (*E*-value ≤1e-5) (Additional file 1: Table S1) to any other of the 107 *Bgh *effector candidates. An unrooted phylogenetic tree constructed by the neighbor-joining method and bootstrap analysis of these 83 protein sequences (Figure 3) supports the existence of small families of proteins.

**Figure 4:**
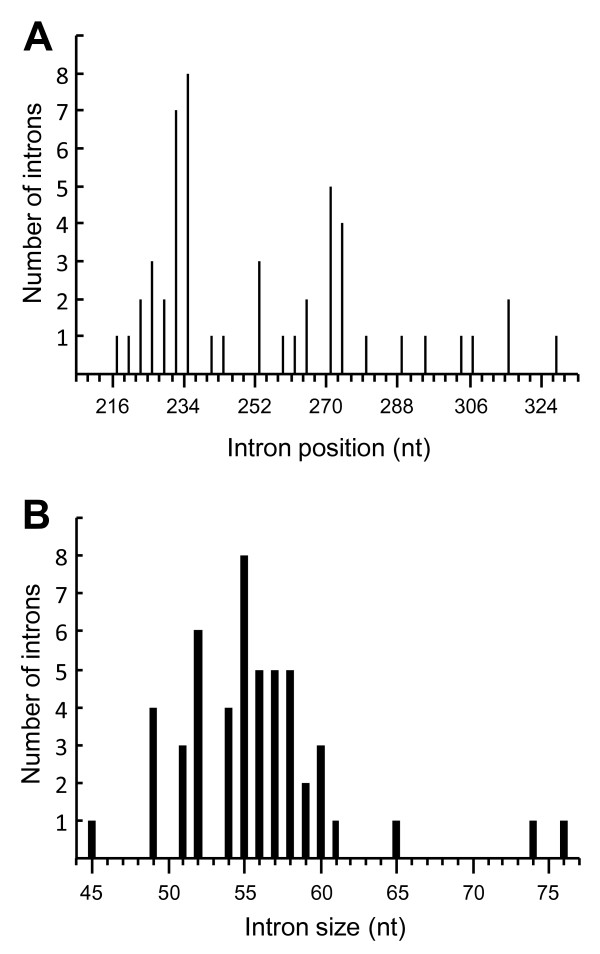
**Shared intron structure in the *Bgh *Y/F/WxC-effector candidate genes suggests common ancestor**. (A) Distribution of intron positions relative to the first nucleotide of the start codon. (B) Distribution of intron sizes. The figure is based on the 51 intron-containing genes for which both full-length EST contigs and full-length genomic sequences exist (see Additional file 4: Figure S3).

In order to further address the relationship of the Y/F/WxC-genes, we studied their intron structure revealed by comparison of the EST sequences and the genomic data available on http://www.ncbi.nlm.nih.gov. Intron position is an ancient conserved genomic character that is highly useful for phylogenetic analyses of genes [16,17]. The study was conducted on the 54 genes in which both a full-length EST contig and a full-length genomic sequence could be established for the entire open reading frame (see Additional file 4: Figure S3). Fifty-one of these had an intron approximately two-thirds into the coding sequence. Closer inspection of these 51 Y/F/WxC-genes demonstrated that the intron is always positioned between 218 to 329 nt from the translation start codon (Figure 4A). Since introns in unrelated genes occur randomly, this narrow window suggests that these 51 genes could be related. Substantial support for this hypothesis came from the finding that the introns in all 51 genes are located in phase 1 positions; i.e. between the first and the second nucleotide of a codon. Generally, introns can occur in all three phases, but predominantly in phase 0 [16]. Therefore, this finding of phase 1 positions in 100% of the genes appears to be highly non-random. Another character to consider is intron length, which also is highly variable in unrelated genes. Meanwhile, these 51 introns had lengths only varying from 45 to 76 nt (Figure 4B). Forty-five of them occurred in groups, of two or more sequences, with the exact same length varying from 49 to 60 nt. These results strongly suggest that the genes encoding the Y/F/WxC-effector candidates originate from a single ancient gene and that evolution has favored numerous tri-nucleotide insertions and deletions, while preserving the Y/F/WxC-coding sequence and the intron.

### Expression of *Bgh *effector candidates is linked to haustoria formation

**Figure 5:**
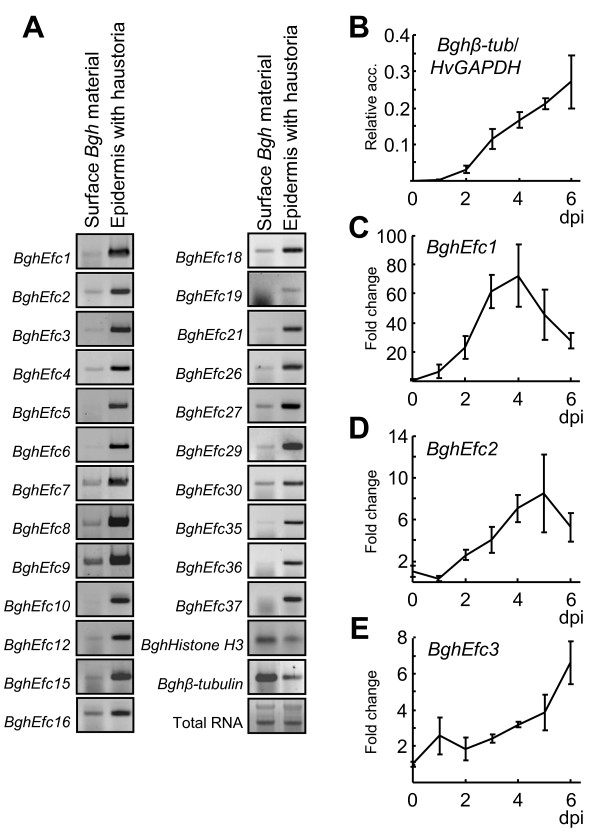
**Transcripts encoding the highest expressed *Bgh *Y/F/WxC-effector candidates accumulate predominantly in haustoria**. (A) Semi-quantitative RT-PCR on total RNA isolated from *Bgh *spores and hyphae collected from the leaf surface 7 days post inoculation (dpi) and from barley epidermal strips containing *Bgh *haustoria (surface fungal material removed). (B-E) qPCR on RNA form time-course of barley whole leaf/*Bgh *material. (B) Transcript accumulation of *Bghβ-tubulin *relative to the barley *HvGAPDH *transcript. (C-E) Transcript accumulation of Y/F/WxC-effector candidate transcripts relative to 0 dpi. *Bghβ-tubulin *used as internal standard.

The identification of the Y/F/WxC-effector candidates in our EST library, prompted us to analyze the expression of 23 of the most abundant Y/F/WxC-effector candidates in different fungal tissues. In order to execute this experiment, we extracted RNA from the *Bgh *fungal material at the leaf surface, and from barley epidermal tissue, containing a high density of haustoria. Y/F/WxC-effector transcript levels were analyzed in these samples by semi-quantitative RT-PCR. As internal controls, we analyzed the transcript level of *BghHistone H3 *and *Bghβ-tubulin *showing similar expression levels in the two samples. For all effector candidates analyzed, we detected a significantly higher transcript level in haustoria-containing epidermal tissue than in the surface fungal spores and hyphae (Figure 5A).

To further characterize Y/F/WxC-effector candidates during infection, we conducted a time-course experiment where barley leaves were colonized by *Bgh *for up to 6 days. Within the first day, limited fungal growth occurs, since this requires functional haustoria which are established within 1 day post inoculation (dpi). After day 1, colony formation starts, and within the time-course the fungus grows unhindered with many haustoria present in the host epidermal cells by day 6.

To analyze the relative transcript level of the Y/F/WxC-effector candidates, we first established *Bghβ-tubulin *as reference gene to monitor the fungal growth in our time-series. This was done by normalizing the *Bghβ-tubulin *transcript levels to the barley *HvGAPDH *transcript expressed in the leaf. Within the first day, the *Bghβ-tubulin *transcript did not increase. From day 2, a linear increase in transcript level occurred through-out the time-course (Figure 5B). This *Bghβ-tubulin *transcript profile reflects the maturation of the first haustoria between day 1 and 2, and the subsequent fungal growth. Thus, the *Bghβ-tubulin *transcript can be used for normalizing gene expression in the fungus during infection. We then selected to analyze the transcripts of the three Y/F/WxC-effector candidates for which the highest number of ESTs occurred. qPCR analysis showed that the *BghEfc1 *and *BghEfc2 *transcript levels gradually increased relative to the *β-tubulin *transcript between day 1 and day 4-5 (Figure 5C and D). Thereafter, *BghEfc1 *and *BghEfc2 *transcript levels declined. The *BghEfc3 *transcript showed a different profile, with a slow but gradually increasing level through-out the time-course (Figure 5E). All together, the results indicate that these Y/F/WxC-effector candidates are expressed in haustoria predominantly during late stages of infection.

### Y/F/WxC-proteins in rust fungi

The discovery of Y/F/WxC-effector candidates in *Bgh *prompted us to search the genome data of the serious wheat stem rust pathogen, *Pgt*, another haustoria-forming biotrophic fungus attacking members of the grass family, *Gramineae*. Using the *Pgt *genome, it was possible to analyze whether the general occurrence of the Y/F/WxC-motif is random. In the 20,566 *Pgt *encoded proteins, Y, F, W and C occur at frequencies of 2.41, 3.62, 1.31 and 1.48%, respectively. In an unbiased distribution, the Y/F/WxC-motif should occur once every 922 amino acid. However, the motif occurs on average every 777 amino acids, suggesting positive selection for the Y/F/WxC-motif in the *Pgt *proteins. Further analysis revealed that 2,830 of the 20,566 *Pgt *encoded proteins have a signal peptide. We then turned to study the N-termini of the mature forms of these 2,830 proteins (from the signal peptide cleavage site to amino acid number 45 calculated from the N-terminal methionine). In these 2,830 N-termini, the Y/F/WxC-motif occurred on average once every 326 amino acids. This led to the identification of 178 Y/F/WxC-proteins, in all of which the Y/F/WxC-motif occurs within the first 27 amino acids from the signal peptide cleavage site (Additional file 5: Table S2).

**Figure 6:**
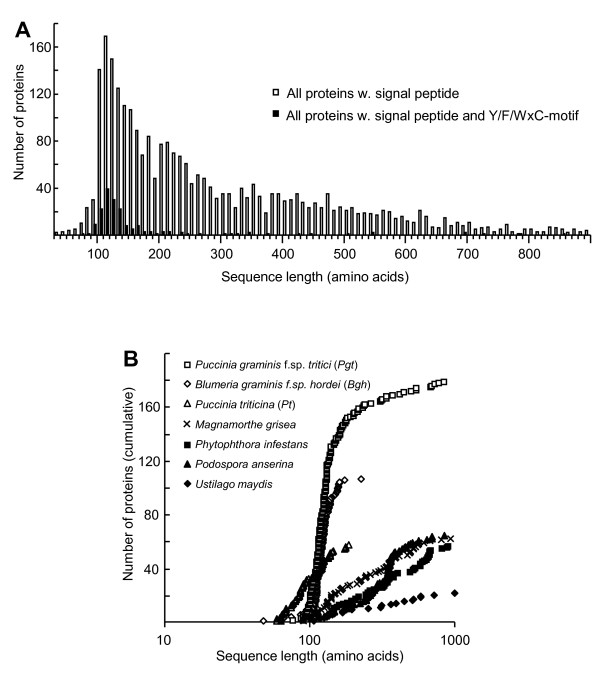
**Only powdery mildew (*Blumeria*) and rust (*Puccinia*) fungi encode Y/F/WxC-effector candidates**. (A) The proteome of *Puccinia graminis *f.sp. *tritici *was screened for proteins with predicted signal peptide, with and without Y/F/WxC-motif, which were plotted against their length. (B) Proteomes of six fungi and an Oomycete were screened for proteins with a predicted signal peptide as well as a Y/F/WxC-motif, which were plotted cumulatively against their length. Only the 105 *Bgh *Y/F/WxC-effector candidates with known lengths are plotted. The proteomes were derived from publically available genome databases and EST library (*Bgh *and *Pt*) databases. Only Y/F/WxC-motifs after the predicted N-terminal signal peptide and within the first 45 amino acids from the N-terminal methionine were included.

We then analyzed how these 178 *Pgt *Y/F/WxC-proteins were size-wise distributed relative to all the 2,830 proteins with a signal peptide. As shown in Figure 6A, there is a striking over-representation of these Y/F/WxC-proteins within the smaller proteins. Twenty percent of the proteins between 90 and 140 amino acids long are Y/F/WxC-proteins, while this number is 2.6% for the rest of the proteins.

BLAST searches against the NCBI nr/nt database revealed that 94% of these 178 *Pgt *sequences had no homologues outside of *Puccinia*. Seventy-eight of these sequences do not have a significant BLAST hit (*E*-value ≤ 1e-5) to any other of the 178 *Pgt *Y/F/WxC-protein (Additional file 5: Table S2). However, the remaining 100 *Pgt *proteins group into 27 clusters, ranging from two to 14 proteins (at least one BLAST hit, *E*-value ≤ 1e-5, to another member of the cluster) (Additional file 5: Table S2). Accordingly, most of these 178 *Pgt *Y/F/WxC-proteins have the same sequence characteristics as the 107 *Bgh *effector candidates, namely that they have signal peptides, they have the Y/F/WxC-motif, they are short, and they are found to be unique to *Puccinia *(Additional file 5: Table S2).

Compared to three other fungi that do not form haustoria, *Ustilago maydis*, *Podospora anserina *and *Magnaporthe grisea*, and the Oomycete *Phytophthora infestans *that does form haustoria, there was an obvious over-representation of genes encoding this effector candidate-type proteins in *Pgt*. This is clear when plotting the number of all proteins with signal peptide, and a Y/F/WxC-motif within 45 amino acids from the N-terminal methionine, cumulatively against their length (Figure 6B). In addition, this result demonstrated that the other four pathogens do not encode high numbers of Y/F/WxC-proteins. The *Bgh *effector candidates (105 with known lengths) were also plotted in Figure 6B, and showed a distribution very similar to the signal peptide-Y/F/WxC-proteins from *Pgt*. Furthermore, the other fungi investigated had low numbers of this kind of protein, and in summary, we suggest that the wheat stem rust fungus has a collection of proteins with characteristics similar to that of the powdery mildew Y/F/WxC-effector candidates.

An EST library of *P. triticina *(*Pt*), another wheat pathogen that causes leaf rust, was searched for presence of Y/F/WxC-effector candidate-type proteins. Using the same criteria as for *Bgh *and *Pgt*, we managed to identify 57 such proteins from *Pt*, ranging from 59-186 amino acids in length (Additional file 6: Table S3). BLAST searches against the NCBI nr/nt database identified no homologues (*E*-value ≤ 1e-5). Using the criteria that all members of a cluster must have at least one BLAST hit (*E*-value ≤ 1e-5) to another member, 20 of these proteins group into 7 clusters, ranging from two to 4 proteins (Additional file 6: Table S3). The remaining 37 proteins do not have a significant BLAST hit (*E*-value ≤ 1e-5) to any other of the 57 *Pt *Y/F/WxC-protein (Additional file 6: Table S3). The 57 *Pt *Y/F/WxC-proteins are also plotted in Figure 6B, which reveals a length vs. number distribution similar to those from *Bgh *and *Pgt*. Like the wheat stem rust fungus, the wheat leaf rust fungus also appears to have a collection of proteins with the same characteristics as the powdery mildew Y/F/WxC-effector candidates (Additional file 6: Table S3).

**Figure 7:**
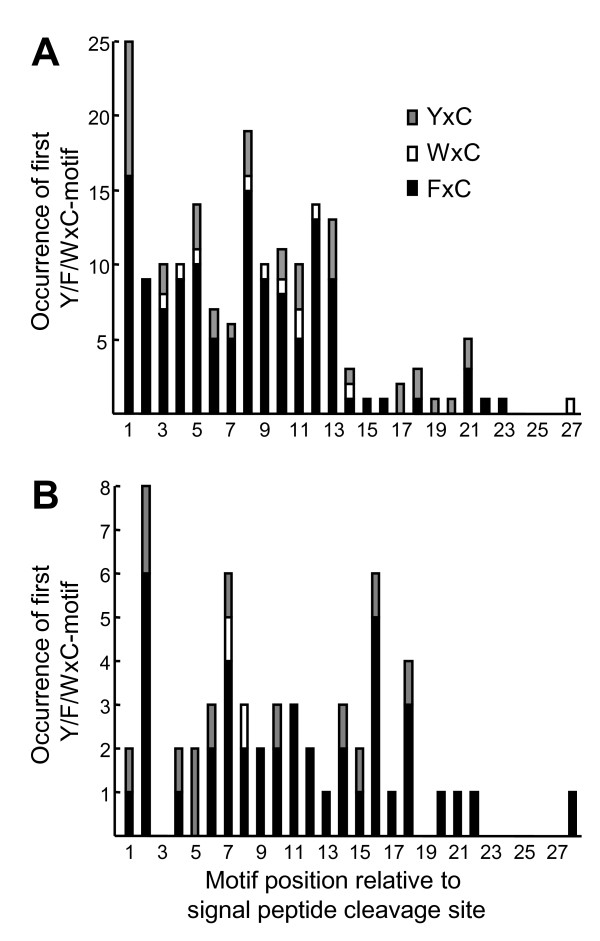
**The first Y/F/WxC-motif in the rust effector candidates is evenly distributed across the N-terminal of the proteins**. The cumulative number of the YxC, WxC and FxC versions of the Y/F/WxC-motif plotted according to the position of the first amino acid of the motif, relative to the signal peptide cleavage site in the 178 *Pgt *(A) and 57 *Pt *(B) effector candidates. Only the first Y/F/WxC-motif in each protein is included.

Figure 7 shows the occurrence of YxC, FxC and WxC and their position relative to the signal peptide cleavage site of the 178 *Pgt *proteins and 57 *Pt *proteins. Unlike the *Bgh *effector candidates, the rust fungal sequences do not appear to have an optimal location for the Y/F/WxC-motif, and while Y is most common in the motif of the *Bgh *effector candidates, F is most common in the *Pgt *and *Pt *sequences. Furthermore, an obvious difference is the number of copies of the Y/F/WxC-motif in each sequence. Whereas the *Bgh *sequences predominantly (87%) had one copy, this was only the case in a minority of the *Pgt *sequences (40%). Two, three and four copies of the motif were found in 43, 13 and 3.4% of the *Pgt *sequences, respectively (Additional file 5: Table S2). In *Pt*, 68% of the sequences had one Y/F/WxC-motif. Two, three, four and five copies of the motif were found in 18, 11, 1.8 and 1.8% of the sequences, respectively, in this rust pathogen (Additional file 6: Table S3).

### Y/F/WxC-effector candidates are shared between closely related pathogens

Effectors proteins are predicted to have evolved in response to appearance of new defense pathways in the host plant, and therefore it is interesting to examine how ancient these effectors are. Whether or not the Y/F/WxC-effector candidate-type proteins are shared between pathogens might shed some light on this issue. Although there is no sequence similarity of Y/F/WxC-effector candidates between powdery mildew and rust pathogens (Additional files 1, 5 and 6: Tables S1 to S3), homologues within powdery mildew and rust fungi can be identified.

While no genome sequence data can be found for other *B. graminis *powdery mildew fungi, there are EST libraries available from leaves that were inoculated with these pathogens. BLAST searching primarily the "EST_others" database on http://www.ncbi.nlm.nih.gov revealed that 38 of the 107 *Bgh *Y/F/WxC-effector candidates shared homology (*E*-value ≤ 1e-5) to proteins encoded by EST sequences generated from powdery mildew-infected wheat (Additional file 1: Table S1). These BLAST analyses resulted in 27 *B. graminis *f.sp. *tritici *(*Bgt*) ESTs, and in all cases, where the sequence was complete at the N-terminal (24 of the 27 sequences), these ESTs encoded unique proteins with a predicted N-terminal signal peptide and a Y/F/WxC-motif. A high level of conservation of the amino acids in the Y/F/WxC-motif between the *Bgh *and *Bgt *homologues was observed. Ten of the homologous pairs shared all three amino acids of the motif. Of the remaining pairs, 12 shared the amino acid at the "Y/F/W" and "C" position, while two only shared the "C". In those cases where the *Bgt *sequences were complete at the N and C-terminal, their lengths ranged from 73-148 amino acids.

Even though *Pgt *and *Pt *both are rust pathogens on wheat, they have rather different life-styles, and they are predicted to be more distantly related than *Bgh *and *Bgt*. Still, we were able to find conserved Y/F/WxC-proteins between *Pgt *and *Pt *(Additional files 5 and 6: Tables S2 and S3). In fact, there are two groups of conserved proteins between the *Pgt *and *Pt *Y/F/WxC-effector candidates. In group 1, consisting of two *Pt *sequences (EC415587 and EC415350) and four *Pgt *sequences (PGTG_16075, PGTG_10961, PGTG_16073 and PGTG_10962), the Y/F/WxC-motif "FgC" is completely conserved. In this group the highest *E*-value was 7e-42. However, in group 2 (*E*-value 9e-46) the three *Pt *sequences (EC402064, EC398870 and EC420434) have "FtC", whereas the *Pgt *sequence (PGTG_03694) has a "YtC".

Furthermore, through BLAST searches against EST sequences in the currently available databases, we have identified homologues (*E*-value ≤ 1e-5) of the *Pgt *and *Pt *Y/F/WxC-effector candidates in a third rust species which infects wheat, the stripe rust fungus, *Puccinia striiformis *(*Ps*). Ten of the *Pgt *Y/F/WxC-effector candidates shared homologies with seven Y/F/WxC-proteins encoded by *Ps *ESTs. One of the *Ps *Y/F/WxC-proteins belong the *Pgt*/*Pt *group 1 above (Additional files 5 and 6: Tables S2 and S3).

## Discussion

Our discovery of the N-terminal Y/F/WxC-motif within a specific position of secreted proteins only from haustoria-forming fungi attacking *Gramineae *represents the first potential molecular "landmark", specific for this group of pathogens. We propose that the Y/F/WxC-proteins serve effector functions, essential for haustoria-forming fungi. This is supported by the fact that 19% of the *Bgh *haustorial transcriptome encode this type of proteins. Furthermore, the finding of conserved *Bgh *Y/F/WxC-gene structure, including intron position and size, contributes to establishing the *Bgh *Y/F/WxC-effector candidates as a protein super-family.

Initially, 107 *Bgh *Y/F/WxC-proteins were discovered from our haustoria EST-library. A bioinformatics approach was subsequently taken to define a further 178 and 57 proteins, with comparable sequence characteristics to the *Bgh *Y/F/WxC-effector candidates, from *Pgt *and *Pt*, respectively. We have evidence from BLAST searches of genomic sequence data on http://www.ncbi.nlm.nih.gov that more than the 107 *Bgh *genes encoding Y/F/WxC-effector candidate can be found (data not shown). In comparison, the *Pgt *Y/F/WxC-proteins are genome-based, and therefore expected to include all of the Y/F/WxC-proteins for this pathogen; although it is important to note that automatic gene annotation is often less precise for small genes. Meanwhile, the 57 *Pt *sequences are from an EST-library of only approximately 29,000 clones. As such, this collection is only expected to include a subset of the Y/F/WxC-sequences in this rust pathogen. These numbers of largely dissimilar proteins are reminiscent of the numbers of RxLR-dEER proteins identified in Oomycetes [7]. The finding of Y/F/WxC-proteins in both powdery mildew and rust fungi raises the question whether the Y/F/WxC-motif have a common origin. These fungi have specialized within Ascomycota and Basidiomycota, respectively, most likely reflecting that the Y/F/WxC-motif has evolved convergently in these two fungal phyla. Furthermore, the fact that the Y/F/WxC-proteins within individual pathogens are highly diverse could suggest that the motif has evolved multiple times. On the other hand, there is a striking similarity in the intron positions in most of the Y/F/WxC-genes in *Bgh*, despite the sequence dissimilarity, which points towards the existence of a common gene ancestor. To this end, it is relevant to stress that homologous Y/F/WxC-proteins could be found between different *B. graminis *powdery mildew pathogens and between different *Puccinia *rust pathogens. This provides additional support for the notion that some of the Y/F/WxC-proteins are ancient and have evolved before separation occurred within these two types of pathogens.

Moreover, the highly diverse nature of both the powdery mildew and rust Y/F/WxC-proteins suggests that many different targets exist in their host plants. This agrees with the fact that the regulation of defense is controlled by a highly complex signaling network [18,19], in which a large number of relevant targets can be envisioned. When these signaling elements evolved during plant evolution, new pathogen effectors may have coevolved. Similar dramatic impacts are believed to have driven other gene amplifications and diversifications resulting in superfamilies with conserved intron-exon structures, but poor sequence similarities [20]. Generally, the Y/F/WxC-effector candidates identified are short and unique to their class of pathogen, suggesting that they are unlikely to have enzymatic activity, but rather interact with host defense components to manipulate their activity.

The highly conserved nature of the *Bgh *Y/F/WxC-motif leads us to speculate that it is involved in a common mechanism shared by the Y/F/WxC-effector candidates. The N-terminal RxLR-dEER double-motif in haustoria forming Oomycete plant pathogens has been documented to confer transfer across the EHM [5,6], a function required by some haustoria secreted effectors. While it is tempting to envisage a similar function for the Y/F/WxC-motif, further studies investigating the Y/F/WxC-function are required. Bioinformatic analyzes of all the powdery mildew and rust Y/F/WxC-proteins have not revealed consistent evidence for other conserved amino acid sequence patterns surrounding the Y/F/WxC-motif.

The importance of the Y/F/WxC-motif, is supported by its presence in several powdery mildew and rust fungi. In *Bgh*, it most frequently occurs at amino acid 4 of the mature protein. Such a position is albeit less pronounced in the rust fungi. On the other hand, the rust Y/F/WxC-effector candidates often have more Y/F/WxC-copies. The preferred first amino acid of the motif is the aromatic amino acid tyrosine (Y) in *Bgh*. Interestingly, the aromatic phenylalanine (F) is the first amino acid in the motif in rust fungi. This could indicate that YxC and FxC serve separate purposes. However, this is most likely not the case, since there are examples where closely related proteins have YxC and FxC, respectively. This was seen for seven homologues of *Bgh *proteins in *Bgt *(Additional file 1: Table S1) and for homologues of one *Pgt *proteins in *Pt*. Tryptophan (W) occurs rarely as the first amino acid in all four fungi, suggesting that this aromatic amino acid is less desirable. It is noteworthy that we do not find that histidine can take the first position. Histidine is non-aromatic, but space-wise similar to phenylalanine and tyrosine. At the same time histidine is quite similar to tryptophan without the aromatic group. This underlines that the aromatic group appears to be essential the function of the motif.

We predict that some of the Y/F/WxC-proteins suppress defense manifested in the host cell when haustoria actively mobilize nutrients to the fungal structures outside the host cell. There are evidences in the literature that the defense mechanisms are suppressed in host cells containing haustoria. This is obvious from microscopy of certain types of multicellular hypersensitive response (HR) lesions in barley attacked by *Bgh*. While neighboring epidermal and mesophyll cells undergo HR in such cases, epidermal cells containing haustoria often evade this defense response [21]. This suppression of defense has also been visualized in double inoculation experiments. When a spore from a first inoculation with a virulent *Bgh *genotype establishes a haustorium, then the epidermal cell that contains it looses its ability to exhibit penetration resistance and HR in response to later attacks [22].

The Y/F/WxC-type effector candidates are in several ways distinct from the previously identified AVR_k1 _and the related AVR_a10 _effectors also expressed by *Bgh *[2,23,24]. No sequence similarity could be found between AVR_k1 _and any of the Y/F/WxC-type effector candidates. In addition, there are indications that *AVR*_*k1 *_homologues and genes for Y/F/WxC-type effector candidates are expressed dissimilarly. Y/F/WxC-type effector candidate genes are primarily expressed days after inoculation and primarily in haustoria (Figure 5). Only a single EST corresponding to a Y/F/WxC-effector candidates could be identified in publically available *Bgh *EST libraries of 2,500 clones from germinated and ungerminated conidia [25] and of ~450 clones from leaf surface hyphae (3 dpi, http://www.ncbi.nlm.nih.gov). On the other hand, no *AVR*_*k1 *_homologous transcripts were identified among the more than 6,000 *Bgh *ESTs in our haustoria library from 7 dpi. Even though AVR_k1 _and AVR_a10 _have been demonstrated to confer their function in the host cytoplasm [2,26] it remains unknown how they are transferred from the fungus to the plant cells. They do not have classical signal peptides or other recognizable elements that can be involved in conferring such a transfer. A possible background for the distinct features of these proteins can be that the AVR_k1 _homologues are secreted from the penetration hyphae and early haustorial structures in accordance with the observation that they suppress penetration resistance in barley [2]. Meanwhile, the Y/F/WxC-type effector candidates appear to be secreted from mature haustoria.

Our bioinformatics analysis of the *Pgt *proteome indicated presence of large numbers of small secreted proteins besides those having the Y/F/WxC-motif (Figure 6A). With very few exceptions, these proteins have no annotated homologues (data not shown). We would therefore not be surprised if also they will turn out to be effectors. Rust fungi endure a significant part of their life in the intercellular space in the host tissue, and it can be envisioned that they need a much wider palette of effectors. Powdery mildew fungi to a large extent live outside the leaf with less direct contact to the plant tissue, and despite our finding of many effector candidates, they could be few relative to rust effectors.

## Conclusion

Our findings suggest the Y/F/WxC-proteins are a new class of effectors, highly expressed in haustoria of powdery mildew fungi. The Y/F/WxC-motif is predicted to serve a general and essential function, possibly in transfer of the effector proteins across the EHM. In addition, more than 200 small, Y/F/WxC-proteins with signal peptides were identified from rust fungi infecting *Gramineae*. Since rust and powdery mildew fungi both are specialized forms within Basidiomycetes and Ascomycetes, respectively, it is conceivable that the Y/F/WxC-motif has evolved convergently in these two types of pathogens. Despite poor sequence identity between these Y/F/WxC-proteins, the encoding genes in *Bgh *have a conserved exon-intron structure, pointing towards a single gene-origin within the powdery mildew fungi. This provides further support for these proteins to have related and crucial roles for the biotrophic lifestyle.

## Methods

### Fungal and plant material

The barley powdery mildew fungus (*Bgh*, isolate A6) was maintained on susceptible barley (*Hordeum vulgare*) cv. Golden Promise. The plants were cultivated on soil in a growth chamber at 20°C under a 16 h light, 8 h dark cycle. Leaves of 7-day-old seedlings were inoculated with conidia from seedlings infected 7 days previously.

### EST library

Heavily colonized first leaves (7 dpi) were painted with 10% w/v cellulose acetate in 100% acetone using a fine paintbrush. Following evaporation of the acetone, the external fungal material was trapped in the solidified cellulose acetate and removed. Subsequently, abaxial epidermal strips containing *Bgh *haustoria, were peeled, frozen immediately in liquid nitrogen and stored at -80°C.

Total RNA was isolated from barley epidermal strips containing *Bgh *haustoria using the Fenozol™ total RNA isolation reagent (Active Motif) according to the manufacturer's instructions. From total RNA, polyA RNA was prepared using mTRAP™ kit (Active Motif) and used for the construction of a cDNA library. The cDNA library was generated using the Creator™ SMART™ cDNA library construction kit (Clontech). Approximately 1 μg of mRNA was transcribed into cDNA. Following ds cDNA synthesis by primer extension, the cDNA was purified using the GFX PCR DNA and gel band purification kit (Amersham Biosciences). The purified cDNA was digested with the *Sfi*I restriction enzyme and size-fractionated by electrophoresis. The 0.5-8 kb fraction was ligated into an *Sfi*I digested, dephosphorylated pDNR-LIB vector as supplied with the Creator™ SMART™ cDNA library construction kit. An aliquot of the ligation products was transformed into *Escherichia coli *TOP10 chemically competent cells (Invitrogen) and plated on LB medium containing chloramphenicol. A total of 1.5 × 10^6 ^recombinant clones were obtained, with an average insert size of approximately 750 bp.

### Sequence processing and analysis

DNA sequencing was performed from the 5' end using the M13 universal sequencing primer (5'-GTAAAACGACGGCCAGT-3') at BGI LifeTech Co. (Beijing, China). Following sequencing, base calling and quality assessment was evaluated by the Phred algorithm [27]. All EST sequences with a Phred score less than 20 were rejected. All sequences were manually trimmed for vector. A total of 11,484 clones were sequenced yielding 9,928 ESTs with an average sequence length of 360 bp. Cluster analysis, performed using the TIGR software TGI Clustering tool (TGICL), revealed 4,327 singletons and 1,196 contigs resulting in 5,523 unigenes. Sequences were analyzed using BLAST searches within the databases maintained by the National Center for Biotechnology Information (NCBI), the *Blumeria graminis *EST database in COGEME http://cogeme.ex.ac.uk/ and the *Hordeum vulgare *EST database in PlantGDB http://www.plantgdb.org/. Similarities were classified as hits indicating homology when the expected *E*-value was ≤ 1e-5. Based on these BLAST searches, it was decided whether the ESTs were of plant or fungal origin. According to the best estimate, there are 3,416 plant and 6,512 fungal ESTs, making 2,337 plant and 3,186 fungal unigenes. Putative signal peptides in the deduced amino acid sequences were predicted according to the hidden Markov models using the SignalP 3.0 Server http://www.cbs.dtu.dk. Sequences were aligned using the slow and accurate pairwise alignment in CLUSTALW http://align.genome.jp/ with a multiple alignment gap opening penalty of 10 and a gap extension penalty of 0.05. Alignments were visualized using Genedoc. The *Bgh *Y/F/WxC-effector candidate proteins were grouped based on BLASTP similarity using the criteria that all members of a group must have at least one BLASTP hit (*E*-value ≤ 1e-5) to another member of the group. The proteins which clustered into groups were subjected to phylogenetic analysis. Proteins for which the full-length sequence is unknown were omitted. MEGA4 [28] was used for phylogenetic analysis using the Neighbor-Joining (NJ) method [29]. A bootstrap consensus tree was inferred from 1000 replicates [30] and branches corresponding to partitions reproduced in less than 50% bootstrap replicates are collapsed. The Poisson correction method [31] was used to compute the evolutionary distances which are in the units of the number of amino acid substitutions per site. Preliminary *Bgh *genome sequence data at http://www.ncbi.nlm.nih.gov were use to obtain full length coding sequences, when these were not included in the EST sequences. Genome sequences were used for intron identification based on comparison directly to EST sequences.

Protein sequences for *P. graminis *f.sp. *tritici *(20,566), *U. maydis *(6,522), *M. grisea *(11,109), and *P. infestans *(22,562) were downloaded from the Broad Institute http://www.broad.mit.edu and for *P. anserina *(10,614) from Université de Paris-Sud XI/CNRS http://podospora.igmors.u-psud.fr/. Of these proteins, 2830, 1,184, 2,244, 3,061 and 1,899 where predicted to have an N-terminal signal peptide (as above) of which, 178, 22, 62, 61 and 65 where found to have a Y/F/WxC-motif within the first 45 amino acids of the of the N-terminal methionine, respectively. EST sequences for *P. triticina *(34,273) were downloaded from http://www.ncbi.nlm.nih.gov. Derived amino acid sequences for 14,843 N-terminal protein sequences greater than 60 amino acids were predicted within all six open reading frames. Of these, 3,444 were predicted to have an N-terminal signal peptide. Further analysis, excluding all but the longest open reading frame for each EST, identified 57 unique sequences with a Y/F/WxC-motif within the first 45 amino acids of the of the N-terminal methionine. The 178 *Pgt *and 57 *Pt *Y/F/WxC-sequences were analyzed using BLAST searches within the databases maintained by the NCBI, and similarities were classified as hits indicating homology when the expected *E*-value was ≤ 1e-5. Both the *Pgt *and *Pt *Y/F/WxC-sequences were grouped based on BLASTP similarity as described above for the *Bgh *Y/F/WxC-effector candidate proteins.

### Analysis of transcript accumulation

Semi-quantitative RT-PCR was performed using the Superscript One Step RT-PCR with the Platinum Taq system (Invitrogen). Total RNA was isolated from *Bgh *spores and hyphae trapped in solidified cellulose acetate (7 dpi) and from epidermal strips containing haustoria (7 dpi) using the Fenozol™ total RNA isolation reagent (Active Motif). To eliminate genomic contamination, RNA was DNase treated using the RQ1 RNase-free DNase (Promega). Primer sequences used for this detection are listed in Additional file 7: Table S4. RT-PCR reactions on 80 ng of total RNA and all primer pairs were run under identical conditions: 30 min at 50°C for first-strand cDNA synthesis, 94°C for 2 min; followed by 25 cycles of 15 s at 94°C, 30 s at 51°C, and 1 min at 72°C; and a final extension of 10 min at 72°C (except *BghEfc30*, *BghEfc35 *and *BghEfc36*, 30 cycles; and *BghEfc3*, *BghEfc19*, *BghEfc21 *and *BghHistone H3*, 35 cycles). RT-PCR products were visualized on 1% (w/v) agarose gels stained with ethidium bromide. The RT-PCR data shown is representative of results obtained over a number of independent experiments. Minus RT control reactions were performed to confirm the absence of residual DNA.

qPCR transcript analyzes were conducted on total RNA isolated from intact barley leaves at indicated time-points after inoculation with *Bgh*. Total RNA was isolated using the Aurum™ Total RNA kit (Bio-RAD). cDNA synthesis was performed using the DyNAmo™ cDNA Synthesis Kit (FINNZYMES) according to the manufacturers instructions. Quantification was performed on a Stratagene MX3000P real-time PCR detection system using the Stratagene Brilliant^®^R II SYBR Green mastermix. The assays were performed on three independently isolated RNA samples, each loaded in duplicates. The *Bghβ-tubulin *transcript level was normalized to the barley *HvGAPDH *transcript level, and the transcripts levels of *BghEfc1*, *BghEfc2 *and *BghEfc3 *were normalized to the *Bghβ-tubulin *transcript level. Primer sequences used for this detection are listed in Additional file 7: Table S4.

### Sequence data

The sequence data from this study have been submitted to NCBI http://www.ncbi.nlm.nih.gov under accession nos. GT883214-GT884367, as referred to in Additional file 1: Table S1.

## List of abbreviations

*Bgh*: *Blumeria graminis *f.sp. *hordei*; Efc: effector candidate; EHM: extrahaustorial membrane; HR: hypersentitive response; *Pgt*: *Puccinia graminis *f.so. *tritici*; *Ps*: *Puccinia striiformis*; *Pt*: *Puccinia triticina*.

## Authors' contributions

DG generated the cDNA library. JE assisted on the EST contig assembly, DG performed all the bioinformatics work, including the discovery of the Y/F/WxC-motif. DG and HB performed the RT-PCR and qPCR analyses, respectively. CP assisted on the bioinformatics work. ZZ and HTC conceived the study and obtained the necessary grants. HTC wrote the manuscript that all authors have contributed to, read and approved.

## Supplementary Material

Additional file 1 **Table S1. *Bgh *Y/F/WxC-effector candidates**. Data assembly related to the 107 barley powdery mildew fungal Y/F/WxC-proteins. Click here for file

Additional file 2 **Figure S1. The Y/F/WxC-motif of the first 35 identified *Bgh *effector candidates aligns perfectly**. Multiple sequence alignment of the derived amino acid sequences of the mature proteins performed by CLUSTALW and visualized using Genedoc. Shading represents conservation of amino acid similarity at each position: residues conserved between sequences are shaded in black. Click here for file

Additional file 3 **Figure S2. The Y/F/WxC-motif of the 107 *Bgh *effector candidates aligns in all but three sequences**. Multiple sequence alignment of the derived amino acid sequences of the mature proteins performed by CLUSTALW and visualized using Genedoc. Shading represents conservation of amino acid similarity at each position. Click here for file

Additional file 4 **Figure S3. Position of introns in the *Bgh *Y/F/WxC-effector candidates**. The open reading frame is indicated in underlining, Y/F/WxC-coding sequence in bold blue, introns in bold red and untranslated regions in italics. Where sequence has been identified exclusively from genomic sequence, it is indicated in white with black highlight. Where sequence has been identified exclusively from EST sequence, it is indicated in grey highlight. Click here for file

Additional file 5 **Table S2. *Pgt *Y/F/WxC-effector candidates**. Data assembly related to the 178 wheat stem rust fungal Y/F/WxC-proteins. Click here for file

Additional file 6 **Table S3. *Pt *Y/F/WxC-effector candidates**. Data assembly related to the 57 wheat leaf rust fungal Y/F/WxC-proteins. Click here for file

Additional file 7 Table S4. Sequences of primers used for semi-quantitative RT-PCR and qPCR. Click here for file
