# Supplementary material for: Powdery mildew fungal effector candidates share N-terminal Y/F/WxC-motif
Source: BMC Genomics. 2010 May 20;11:317. doi: 10.1186/1471-2164-11-317 (PMC2886064; doi:10.1186/1471-2164-11-317)
Supplement: Additional file 2 — Figure S1. The Y/F/WxC-motif of the first 35 identified Bgh effector candidates aligns perfectly. Multiple sequence alignment of the derived amino acid sequences of the mature proteins performed by CLUSTALW and visualized using Genedoc. Shading represents conservation of amino acid similarity at each position: residues conserved between sequences are shaded in black. [file 1471-2164-11-317-S2.PDF]

```
               *        20         *        40         *        60         *        80         *       100         *       120         *       140         *
BghEfc21 : ------------ADYRCDNGATIPARYITSALARVREQAQNGEDGF---------TLQQPGRPYKATPTTRSSIQLYWTHIDVDRNQSYVNGEYYGYEVVYSSNFDLYYVNAFKATDTWKENLISCQ------------------------ : 106
BghEfc34 : ------------ADYICDNEAEVPAKFVNDFLSHARVQARNRQSGY---------SLENPGDVYSGGRNP-----LYWRAIIVSQDLERFNGQHYEYRIVYDSQFNLHKLEAVRLNGGGSETTFHCYSY----------------------- : 103
BghEfc28 : -----------FGDYSCSNDMVISGREIDAAHDKAKSYINNAASYS---------RYLN-TILYNGNLNSEPNSVYYWFLAQLKTDQRNVTTTYYLLTSTTGEFLGVISTGDYQDIG---TSDTWCSPY----------------------- : 105
BghEfc29 : -----------FGDYSCSNDMVISGREIDAAHNKANSYINNAASQR---------MYLS-KIVYNGNLNSESDSVYYWFITQFKADQRNVTTKYYLLTSTTGEFLGVISTGDYQDIG---TSDTWCSPY----------------------- : 105
BghEfc15 : -----------LDGYRCV-EQNISREILMREVNQSYGEAVEQDPGN---------AFFRDSTLSANLEFS-----FTFPSYND---------PNSTIKVFFNNRKQILRVQTFLFGQIHDCHEIAKWHA----------------------- :  94
BghEfc36 : -----------LDGYNCL-GQVLSHDLLTEEINLSYDKTVPQDPGN---------EFFTDSSISVPLVFS-----ISFPQPDY---------PLTKVKILFNHRKQIIAVQAFAWDKYFNCEEIIE-------------------------- :  91
BghEfc17 : --------NSGNAKYNCS-GVIFYSESINASTAMASTINIGSLNGY---------PAPYPVYGLSGTAPY-----HLFPMVKDVMVYAGGVVSKFFLIIDINDIEQGMVYVREGYEGYLPCTAM---------------------------- : 101
BghEfc24 : -------VNSGYPMYNCR-GVTIYSDSVINSAARAHGLEIGEINGY---------PMDMDPYGVNGPPPH-----KSFPLVPDQEVYSGGIESFYFVIVDGNGQHQGVVYSAVG--GYVPCQDLVNPEY----------------------- : 105
BghEfc4  : -----------NQHYKCDDGDPVGVEEAQSMEDDCASTKNLDEHPA---------ILTGESHKSYLFARR-----------------VYLNKPGISIYMIQVYGNPKKYQLSRHVDKEWIVCSLQDIR------------------------ :  91
BghEfc11 : -----------NLYYKCDVGDSVNLEEVLNMDCDAALTENRDEHPR---------IPTGESHKSYFFTKR-----------------ACRDRLGLACYLLQVYGYPKKYQFSQYSNMEWKVCSLQDIR------------------------ :  91
BghEfc19 : -----------EQYFKCHSGRQFTMERVRTYANYATTRLSQTIEPT---------VDEAETRVSFEFD------------------LLDSRNEKYYYLVQFSSSESTYYVFELGGTYWQPCSFHRN-------------------------- :  88
BghEfc22 : ------------SYYECNSGQIFDISTVRIKAASATHEQSHTIEPD---------VPSPRERKSFAFD------------------VVNDQYGSQWYLVQYTGTFPTYYFLRVGWHRLEPCTFRRSNSVEIGAGCNETSLIWNVERLFLFGT- : 113
BghEfc27 : -----DDLEDGYVMYNCF-GKKFYSENVLNSVTEAIKHKRAPIRGY---------PQSFNADCYGGLPPY-------TIFPVPDIERFDIKTSGLYFFVVMDSRNAKVGMAYKTTHGHEPCKDILLQ------------------------ : 105
BghEfc37 : --------APGEYVYACGDSDLFTIKEAQNYAVEATPNGRYESDPD---------SSGHDVLRAHHLCRVN-----------------KYEESGYYVVRAVKDRLALELWKYVDGRWMPCPILSVDS----------------------- :  93
BghEfc5  : ----------ADPYFECSMNTAVSFSGIIFYEQSHEYLDAEPGDPE---------GPNGEIYPARRFTRVR-----------------RDGSDVLILIQSLDEYPLRRAYEKTEQGWRLCPFHKP------------------------- :  89
BghEfc30 : -----------LMTYECVSKNTIPIATIEKYEKKATVEKAKEGDPE---------VPRGQRCKAKRFTVR-----------------LPNRETMLYLLQVIGPQPSYQLYEYHEPKWHPCLLRQGTWLAS--------------------- :  93
BghEfc9  : -----------DFVFNCY-GVEVTSAMIQRTYLLSLGGRVPNYPEI---------TSISYLYNNEPIVIFP---------ILGDDETSIAAELYRPFLIWSQSFLDWGVYFLGDNGYEICYKNPDYNGYTFRQIAFRNL------------ : 109
BghEfc20 : ------------VTFNCSGYKIDESVVAREMKKRIRQNDLSQSTLG---------SHSTTFYKYFDITTSG-----------------LTSSSSTGFRCYTNTGTNSPDVREWDGVSWTPCSQE---------------------------- :  86
BghEfc3  : --------------YNCE-GAMFSEDYVRQTLHRAVARFVPEAGSH---------PNADVEAEYLYFPLLS------------SGQLWDREQPIENYFIKCHRASRDWKVIILDNPGYHAGREENSNSRECSVIQ----------------- :  99
BghEfc14 : -----------QNDYECSNGVVVEGDVIQAALNDISTDSPSTLYAA---------GTLYVNNEYLNRWWKEMT----------VPESYHVGDTETSYRLIFDDNKIVRSIQLVYHFRSGIQISQCTEV----------------------- :  98
BghEfc10 : -------------GYLCP-NSFISDELINSVKNFVQSHWGHQDLVH---------FKNRLGKDRWRIHIN---------------TIPPGHPGTSNSYGIFNSNQELVNVVERTGRGYRDCDIH---------------------------- :  86
BghEfc18 : -----------MIGYQCD-RDIIPSELVENAIRGSYKYLYEEQYDK---------LINFDVEDKINVVLS-----------------HQSRFGEDYSFWASHNKWGDLIGVFYRDGTFDRDCYTVPFN------------------------ :  90
BghEfc12 : -------------YWDCD-DYRVPDKNVRDAAVFAFSKEKGSFHGY---------PITVHAASALSRLGSIR-------KFPVDCSEENWQGEHVNFYVLTNRDRKFIQVVYTFGDGGNCAHVQD-------------------------- :  95
BghEfc6  : -----------LEYFDCQ-GVTFTLDYVNRAAYQALEMQMYSHHDF---------PIPYNDNGMSRGREGYR-------QFPLLTSGEIWNGEQFDYYLLTSPELDTLDVFSTANGNVACDVVNG------------------------- :  97
BghEfc2  : -----------WIYFQCPSNSSILFELVMGRAREIYYKVNAEGATI---------QPGQNMLGGTAIWGSMED-----GDLFYEVPFVPDFKTTNKYKISINAASTEIYLRETTQDSTGDCSTGPPENHPPSNKVTDWLWKRY---------- : 118
BghEfc7  : --------IILDGVYRCPGLGYDLDANEVAQKVDSININAEKADWY---------DFEENGVKYQNHHLFG----------------EYQTSDEKDVVIAVNEDKSSVKVSVFRGKELLDCQHIPAEDAVKSKGRTSKGRTSKGSPSRAARR- : 119
BghEfc8  : -----------KSNYVCPGGYEIAEEAVQAAAEKVDRHPKKTQDSIRK-TENTLHITFDLDNKTIDDTEYSISARIFTPDNTIRVFETETKSGKQGYCGLIAVNEEDDLSYDESFEAELSDDDSEDADLSNDDDDDDDDDDDEEEVAEGKPKTH : 142
BghEfc31 : -----------EKIFSCECGYQILESDVIAAARNIDLDAKIYKQEAS--------NTDRDQSRFQLDAVT-----------------YADGLYQISAIIYIQSMTVRVIQVKYRTRQEKFCDLIEGHA----------------------- :  92
BghEfc23 : ------------ATYECPFGRYFTSDDILAASSSRSAPDKVTYANG--------HAFNWYFLNRYTHNDEFVEVHGLLDTTTRKVVPIASFAGRNWRLCSLGQRSDAQNANDLSDFANNHFDYSYMTAEIENALSD--------------- : 116
BghEfc26 : ------------RTWQCRSGDILYEEDVYSHTNMNSFEHFPFKTIED--------EVYENTSLSRGVAYK-----------------VLDVHPSSSSATTFAVFTSNKRVAVVYQQGYRNNRKFIEECT----------------------- :  92
BghEfc16 : ------------SYYDCNGSHISISKVHQAIHNYGNFQQNHGYLLN---------SFRLKHTNTATIDYID-----------------PRHQAYTFLVDFDQGGGIKRVYSALGREIDECKYMY-------------------------- :  86
BghEfc1  : -----------AKDYTCGGVLIPGSRIEEEVETKKNSLQVVYYKQY----------LRDEEYGRVNFNVDQP-----------------AEGSSPLTVEVAFNLMGVVLGITATRDNVSVDCKKEV-------------------------- :  88
BghEfc35 : -AFKGYHIDEGLKSFDCDGLVISEPDYRDQQYRASRSIENNVHPHQTTGDFMNAYLGNFSDGRWVIFSDESSRRFYLCNLDIPGTIEIADNKSQETSYVLVLDNLGRAVAIFTKLTVTDTDMSGESLTGTKYEICGIVR-------------- : 138
BghEfc13 : AHYQLSDIQDSQKRFSCYDQIFDYNGLRDDQQNAYARLRQDLLSEITIP-----EIEAKLGNLRYGSKVMYKSSVPNSRYYQFSTEGDGDSNYFYTYILIIDSHGHISAVLGRITTVQSVAPSLRISETFCTII------------------ : 129
BghEfc25 : -DYKSPHINEGEKSFKCNKKIINASQYWEEKLLADNLMRQRQKIIVQAQG-FYDVFWSRRETNLVLYDDNTASYFFDHKLTSIADVRKSRKFEFEIRYTLVIDNFGRVCAMFMNEQVVGEAGSSLKPELKPRYTKSFCTINS----------- : 140
```

Figure S1. The Y/F/WxC-motif of the first 35 identified *Bgh* effector candidates aligns perfectly. Multiple sequence alignment of the derived amino acid sequences of the mature proteins performed by CLUSTALW and visualized using Genedoc. Shading represents conservation of amino acid similarity at each position: residues conserved between sequences are shaded in black.
