# Supplementary material for: Powdery mildew fungal effector candidates share N-terminal Y/F/WxC-motif
Source: BMC Genomics. 2010 May 20;11:317. doi: 10.1186/1471-2164-11-317 (PMC2886064; doi:10.1186/1471-2164-11-317)
Supplement: Additional file 3 — Figure S2. The Y/F/WxC-motif of the 107 Bgh effector candidates aligns in all but three sequences. Multiple sequence alignment of the derived amino acid sequences of the mature proteins performed by CLUSTALW and visualized using Genedoc. Shading represents conservation of amino acid similarity at each position. [file 1471-2164-11-317-S3.PDF]

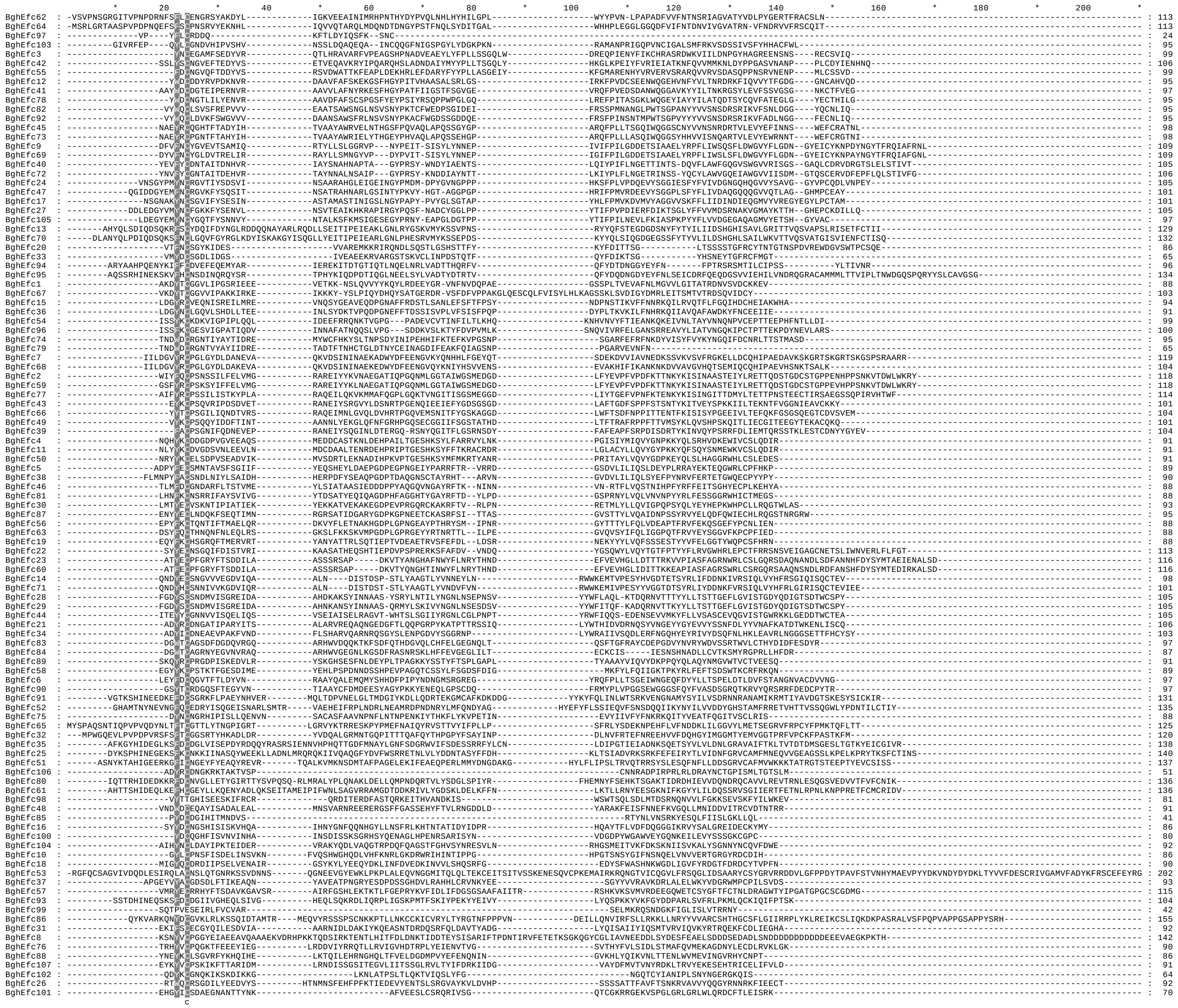

Figure S2. The Y/F/WxC-motif of the 107 *Bgh* effector candidates aligns in all but three sequences. Multiple sequence alignment of the derived amino acid sequences of the mature proteins performed by CLUSTALW and visualized using Genedoc. Shading represents conservation of amino acid similarity at each position.
