# Supplementary material for: Powdery mildew fungal effector candidates share N-terminal Y/F/WxC-motif
Source: BMC Genomics. 2010 May 20;11:317. doi: 10.1186/1471-2164-11-317 (PMC2886064; doi:10.1186/1471-2164-11-317)
Supplement: Additional file 4 — Figure S3. Position of introns in the Bgh Y/F/WxC-effector candidates. The open reading frame is indicated in underlining, Y/F/WxC-coding sequence in bold blue, introns in bold red and untranslated regions in italics. Where sequence has been identified exclusively from genomic sequence, it is indicated in white with black highlight. Where sequence has been identified exclusively from EST sequence, it is indicated in grey highlight. [file 1471-2164-11-317-S4.PDF]

Figure S3. Position of introns in the *Bgh* Y/F/WxC-effector candidates. The open reading frame is indicated in underlining, Y/F/WxC-coding sequence in bold blue, introns in bold red and untranslated regions in italics. Where sequence has been identified exclusively from genomic sequence, it is indicated with black highlight. Where sequence has been identified exclusively from EST sequence, it is indicated with grey highlight.

>*Bgh*Efc1

atgtttgattgcatattgcagaagtgatggggcaaatggagataaatcttgttcaaacataatcacttctcattcactca
tgcgtataaaggggatcattcccacctcagcactcatattaagataactattttcaaggcagctttcagtccaactcgaa
*gtatag***attgaacatatttatctagctaattcaccaagtacaaatatacag***atccaaaatttcataagcccaaccttcaa*
*c*atgaaaaacctaagctttgtttcgcttgcgcttttcttaagccatttgatgcctgtactggctgctaaagat**tatacat**
gtggcggtgtccttattcctggaagtaggatcgaagaagaggttgaaacgaaaaaaaatagtctgcaagtagtatattac
aaacaatacttgagagatgaggaatatggtagagttaattttaacgttgaccagccagcggaag**gtttgccggaatcgtg**
ccaactagttgttatctcctactgacacttgataacaggaagttcaccgttaacagttgaagtggcatttaatttgatgg
gagtagtcctagggataacagctactcgggataacgtatctgtggattgtaaaaaggaggtgtga*tgagttcccgcgagt*
agtttgatttcttgttcgtcccggccatgatagtggtacttgcatcgtataccaagagttattctcagtggatcatgca
ctgttccaaactgattgaatatgtagttatctattttgaatttggatttgatctcattatccggattcggcgccctacg
ggcccatgtgttacggtttcaccgttgtttgagcttaggcggacacctaagcctaattttgggaattcttacgatgaggt
catatggggtaggtgaagctgaacgaaacggcagtacttgtcaggtggctgaagcgttattacaacttagtgacaactg
gaaatacgtggctgggtaagaagtattgtgcctaacgacaagtcactagtctgtagattca

>*Bgh*Efc2

atcctaactccgtgctaatatcattgagactcatgatgcgatggagacaatactttacaaggagcacccttcactctctc
ccatgcagtacatccttttgtatctttatttagttgagttttcctgccaatttatatccagtagccgtattggttgatag
gatttttggcaacaatatcccacgacatattggcacctataagaccgacagtgaatctcctccggtactaattcttcaa
gtgataatatttacacaactgtaaaaatttctcagcatatttcttcactgacacacccataaattcaattcgtcacgac
*ttgagggcccaagaaagagctacgcaacacttttcttcgacctgaccgaaagccttgttggaggcactttcaag*atgaaa
tttttatgtctgactttcatgtcagtgctcctcagcttctcagcttcagtaaatgcttggatatat**tttcaatgc**ccaag
taatagttccatcttgtttgagcttgtcatgggaagagcacgagaaatttattacaaagtgaatgcggaaggggccacaa
tacaacctggccaaaacatgctcggtggaactgctatttggggtagtatggaggatggagacttattttatgaagtcccc
ttcgttcctgactttaaaa**gtaagtaaatatccaactatttaatacggcggctgttaatctctttcaattataag**ccaca
aataaatataagatctctataaacgctgcttctacagaaatatatttaagggagactacacaagattcaacaggagattg
tagcacaggaccacctgaaaaccatccaccatcgaataaagtgactgattggttgtggaaaagatattag*gcaaattgtc*
attgtaggagaacaagctctaggacaaaagtgtggcgcgagacattttattttcagaccgtagtggctctaagtattgtg
gttataaataatcaattgattttgaatttggattcatttaagctgatttggcgccctatgggcccatgcaggtggctata
aacctatctatatatttacataggaggaaaccttacggtgaaaagcacccaagctttctgatttaattcttaaagtttgc
aatctatcctgcagaaaaaaaaaaaaaaaaaaaaaaaaaaa

>*Bgh*Efc3

ctctctagcttttcttcctacagaaaaatctctcatttatatcgcacgagtctccaaacaacagtttcttttcactcca
aacgcaaacatcacagctcatcagtagattccaaatcttggatttccattgaaaagttaaataatccaaaataaatcttc
*gac*atgaaatcaatcaaccaagcgtggatagttgccatcctaagtttttccgagtttccgctgctttgtca**tataactg**
tgaaggagcaatgttttccgaagattatgtgagacaaactctgcacagagctgtcgctagattcgtccctgaagcaggct
cccacccaaatgccgatgttgaggcagaatacttatactttccactccttagtagtggacagttatgggataggg**gtgcg**
tcttctctgtgcccaatacactttagcgtctaactcttgaaaagaacagccaatagaaaattattttattaaatgtcata
gagcaagtagggattggaaagtaataatcctcgacaacccaggctaccatgcagggagagaagaaaattcgaattcgcga
gaatgcagtgtcatccaatga*tcaaagtaggtatcagcttactagacgcttgacctaggacaggtgaagaaaccagtgat*
aggaatctcatagttcactagagataaatctaagcttcatgacattggtcacgaaaaaaaa

>*Bgh*Efc4

aaattaaatctcgtcagaaatataacttcaatggagccctggtctctactacagacgtcgtcgacattttgacaaattc
atctttgactattgccgacataaacgttgcagtagaacatcgaaaaaaggttaataatcgagactcatagctgctacttc
ctatctgcaaagctttgaacttgatatcagacggctcagcgtcccgagttcgcatttagatttcttggaaccacataccc
*gaa*atgaagtttatcagcaccgcgacaaccgcagcattggccggtatcatactgctggcaccggccgcttatggaaacca
acat**tacaaatgt**gatgatggcgaccccgtgggcgtggaagaagctcagagtatggaagatgattgtgcttctacaaaaa

accttgatgaacacccggctattctaaccggggaatcacacaagtcttatttattcgcccgaagagtgtatctaaata**gt**
tagttttcttgctactctgacatttgtgatattgaccgttccttccagaaccaggtatatctatttacatgatacaagtt
tatggcaaccctaaaaaataccaactttctcgacatgtcgataaggaatggatagta**tgttctctgcaagatattcgtta**
g

>*Bgh*Efc5
actcgctgagagtagatttcctcagatatataatcgcaaggttttgtccacagtctgcatcacccagacagtcgaccaa
ccttccatagactccatcgacagaaacattggcgccgaatactatcaacaattttccctcgaaaacgctttgataccttg
ctatccatcatccattgagcctcaccaataaaatcgagtgtctggctgttcggagaccagattccagatccgtgagacga
*aattctcgaa*atgaagtttctcagcgctgcatcgatggccacattgtctggtcttctattgtcagcgacagctcatgctg
cggatccatac**tttgaatgc**agtatgaacacagcagtctctttctcgggaattatattttatgagcaaagtcatgaatat
ctggacgcagaacctggcgatcctgaggggccgaatggggaaatttacccggcacgccgatttaccagagtcaggagaga
tggaa**gttagtttcccttcctctgacgctgctatcactaaaatctcatgtcttcggccag**gcgacgttctaatcttgatt
caatcattggatgaatatccccttcgtcgagcttatgaaaaaacagagcaaggttggcgactatgtcccttccataaacc
ctaa*taagaggcaatacatcggcgtattgtttttgtcatggcaagagcttatactagaagctc***gtgagttattccttcg**
cattggaaaacaaacaagaaaatcgtctgctaattctgttttctcttcttccag*gaccagctttatttcaacgcactct*
tcacgtctcaataagtacctcccgtaccttcggagataaataccaagagcttagctcggttagagtagtgtacatccatg
atgggaagttcaatacgatgtgactgaggatcctggacaggatagttttgatttcgttcagtttgtcatggcaccaggca
attgacagcctccgcactccaatgggagcttgggtaggagtgggatcgggcgatatttagctaactatcatcgaagtata
tgaacttcgtctcatctccgaatccaaccatgtgttgaacatg

>*Bgh*Efc6
aagtaccaaaatttctatccaaattatctccctatataaagagcttaaatgacaccaatttcagatgtacaaagatttta
tttataagtcatcagtattattgaaatatacttctttggttgttaacattaactttagacccacacattttaaatagtcg
*accttatattcacaaccttattttaagtctttaaaactagatattttcaaa*atgaaattaattaaaatttcatctgcttt
tttagctagttgtatttttgaaactattatagctctagagtat**tttgactgc**caaggtgttacattcactcttgattatg
ttaatcgcgcggcatatcaagcgctcgagatgcaaatgtattcacaccatgacttcccaataccttacaatgataatggc
atgtcaaggggtcgggagggttatcgccaatttccacttttaacttcaggtgaaatttggaatggagaacaatttgacta
ctatctcttgaccagcccagaattggacacattagatgtcttttcaactgcaaatggcaatgttgcatgcgacgttgtaa
atggctga*tgaagaagagctagattggatgaactattcaatatatttgtaagacatgtactgatattgcattcctgaatt*
caaagactagattg

>*Bgh*Efc7
tacacagaagtctattatttaaatgctctcggagttttgggattccataagtatggctgttccctcctcctcgttcgaat
attgcactacctgataagctacatcattcaagttgaatccaccttcatcttacaatcttcgcgtcgttattcagctgtcc
*aaagcagaattatcgtggccgtgctcgtctgaataagaataatacctaaatcgattaaagttttcaca*atgcagttcttt
cgttcagccttagttctatcgctttcgatgctttcagtctcagccatcatcctcgatggtgta**tataggtgc**cctggtct
tggatatgacttggacgcgaacgaagtggcacaaaaagtcgacagtattaatattaatgcagagaaggcagattggtatg
acttcgaagaaaatggcgttaaatatcaaaaccatcatttatttggggaatatcaaacctcag**gtctgttgcccttgtta**
caaacacaattagaggcttccatcactaatatagatcagacgaaaaagatgttgtgattgcggtgaatgaagacaaaagc
tcggtaaaagttagcgtctttcgcggtaaagaattattggattgccaacatattccggcagaagatgccgtgaaatcaaa
gggtaggacttcaaagggtaggacttcaaagggtagtccctcacgagctgcgaggagatga*aatcggagtgagagataga*
catttaaacctcatttaacagggctcaaagattaagtaatgaaatgtttacaaaaaaaaaaaaaaaaaaaaaaaaaaaaa
a

>*Bgh*Efc8
acaatggaaagataagtacttaatgggctcatgcctctcccttcaccacctcataccacattaattccagctctctacgt
*cgcgagcagctcggccgcttaataacaacctttccaccctcagtcacaccattacactccactaaacgtctgcgcaatc*a
tgaagctcctacagtccgcttccatcatcggcctattttccttggccctagccaaaagcaac**tacgtgtgc**ccgggcggt
tacgaaattgccgaagaagccgtccaggccgctgccgaaaaggtcgaccggcaccccaagaaaacacaggactcgatacg
aaagaccgagaatactctgcacatcacgttcgatctcgacaacaagacgattgatgaca**gtctgttgcctctcccctcca**
taccaatgcacagctactaacacccagcgtgaagccgaatattccatctcagctcgaatcttcacgccagacaacaccat
ccgcgtatttgagactgaaaccaagagcggaaagcaaggctactgcggtctcatcgccgtcaacgaagaagacgatctct
cctatgacgaatccttcgaggccgaactctccgacgatgactcggaagatgcggatctttccaatgacgacgatgatgac
gacgacgacgacgacgatgaagaggaagtcgccgagggaaaacccaaaacacattag*actttgggatttccaagagggaa*
tctgacgggcgtcgagcgtaactttcgtgtgttattattcagggctaggtaaacagacgttgctgcgaagggccgaattg
ccggggt

>*Bgh*Efc9
*Agcagctgctaaaaagttcttccatctcatacgtccaattgattgttcgttgcatccggaactttctcgagtctag***gtaa**
gcgcagctcatgcattctcttcttcctctctacctcacactagacatcataaacgggcacagaagctgatggaacatgca
ataag*atctcgcacataagagcaccgtctacacgcttgcaaa*atgaagattcaaaacatgaagtggttcatagcgttcat
gtttacaccaaaatttgcatatgcagattttgtc**ttcaattgt**tatggtgtggaagtaacatcagcgatgatccagcgta
cctatttactttctttgggcggacgtgtgccaaactatcccgaaattacatcaatttcatatctatacaacaatgaaccg
atcgtcatctttcccatccttggcgatgacgaaacgtcgatagcag**gtaactttcttccacctttgattaagctttctga**
cttggacaaagctgaactgtatcgacctttcttaatttggagccaatcttttctagattggggtgtgtattttctgggtg
ataacggctatgagatctgttacaagaacccagattataacgggtatacttttagacagatcgcgtttaggaatctatag
ggcaaccaaaataaaagttctggtgcacgtccgtaatgctgaggtgcatgt

>*Bgh*Efc10
acttaaagtcgaaagttgcacctagatataaatggtttgtgaataacatctatgttttgaacctgataaacttttaaaac
tattcttttcaacagaaatttggtaaatatacatattaaagtaattaaaacattacaataaggaaaaatacttctttcat
*acct***gtaagttatataacgacttttaatttaataccaacaattaagaaaattaacatctaatacatactaattatttgtt**
ttgtag*accagctagacc*atgaagattatcagttcgacaacgattattctcgtcacgagcaatataatgctaccaattgt
tgaggctgga**tatttgtgt**ccaaacagtttcatttcagatgaacttataaactcagtaaaaatttgtgcaatctcatt
ggggccaccaagatctggtacattttaagaacagattaggtaaagatcgttggcgcatccatattaatactatacctcct
ggtcatcctg**gtacgcgaccttaaatcactaatctttctacaactgctaacatatctgttaattcatag**ggacgtcgaat
agctatggcatctttaatagtaaccaggaacttgtaaatgtagtggaaagaaccggcaggggttatagggattgtgatat
acattaa*agacaag*

>*Bgh*Efc11
cagattcagatatgttcaaattaaatctcgtcagaaatataacttcaacggagccctggtctctactacagacgtcgtcg
acatttttgacaaattcatctttgactattgccgacataaaagttgcagaagaacatcgaaaaaaggttaataatcgaga
ctcatagctgttacttccattctactaagctttgaacttgatatcagacggctcagcgtcccgagttcgcatttcgattc
*cttggaaccacattcccgaa*atgaagtttatcagcaatgcgacaaccgcagcattggccggtatcctactactggcacca
gtcgcttatggaaacctatat**tacaaatgt**gatgttggcgattccgtgaacctggaagaggttttgaatatggattgtga
tgctgctttgacagaaaaccgggatgaacacccacgaattccaaccggggaatcacacaagtcttatttttcaccaaaa
gagcttgtagagata**gttagttatcccgctcctacgacattagtcaaattaaccgttcctcccag**gactaggtttagctt
gttacttactacaagtttatgggtaccctaaaaaataccaattttctcaatattccaatatggaatggaaagtatgttct
ctgcaagatat**tcgttag**

>*Bgh*Efc12
atcctatccccttcagaatatcatttctcgccaatccgcaagtagcttcgccccgcatacaatttctgaaatcgttccca
attaagaatacctattaaatttcttgaagacaaggtcgtctcagggacaagttttccgcgactcaaatatagccagcttt
*cgcaaaagagaatcagacaatcacaggactcttcaggagcaccagcaaa*atgagaccattccaacttctgcctgctatta
caatcttcattagtttggagatattaggggttgcaggttat**tgggattgt**gatgattatagagttccagataaaaacgtt
cgagatgcagcagtatttgcctttagtaaagaaaaagggtcttttcatggttaccccataactgttcatgcagcatctgc
attgtctagattagggtcaatacgaaagtttccagtagactgttctgaagaaaactggcaag**gttcgttatttggttcta**
tttcactgaagaaaaatacgctaataatgttggtaggagagcatgtaaacttctatgttttgacaaacagagacaggaaa
tttatccaagttgtttatacatttggagatggcggtaattgtgctcatgttcaggactag*gctgggcttttgtggcagt*
aggactgattctcatctcttggccaatagagagattacaaatctactcagtgaacaagggtaatatcaggtcaagtgtga
aagtctaggatatctggagcgggcattgtagctgtaaagcgtcatagattcccatttcaaaattctccatagaaaatt

>*Bgh*Efc13
agaactatttggatgagtacttatatgagtcttccttctttgatgtatagctatcctccaagtctcactttcaacttcat
*caagttcatctcaatcgatttaacaaatcaaaaactatcatc*atgcgcttctataccgtcgccctccttctccagagcgt
cagcttctctgtggcaaatcttgctcattaccaactatctgatattcaagacagccagaaacgt**ttcagttgt**tacgatc
agattttgactacaatggtctaagagacgatcaacaaaacgcctatgctagactaagacaagacttgttgtctgaaatt
acgattccagaaattgaagcaaaactcgggaatttgcggtacggaagcaaagtgatgtataaaagttccgtgccgaatag
cagatactatcagttttcaacagagggtgatggagaca**gtaagtttttccagtcattaagagagccctatatgttgctaa**
taagtatccaaaggcaactattttacacttacatacttattatcgactcacatggccatatatctgccgttttggggag
gattactacagtccaatctgttgcaccaagccttagaataagtgaaactttttgtaccataatatga*tgatagtaataac*
cataagtctcggagagcttatctggcaattcagctctttatcatcaacctagcctggaacaggaagagaattatgctgaa
tgtctggctgatctgagattttggcaaggtccgtaaaattgttgt

>*Bgh*Efc14
aaacacatcttctctattgaaaactcaaggctacccagtgagccatccatcctatctctcatctagttcgtgcacgcgtc
*gaccgctcacttttcccttcacatccgaaacttcaaac*atgcgcacatccactgtactgagatgcctttttacttggctt
gtcactgctataacgacagtacaatgtcaaaatgac**tatgaatgt**tccaacggtgttgtggttgagggcgatgttattca
ggcagctctcaatgatatctcaactgactcaccgtcgaccctctacgcggcaggtacattatatgttaacaacgaatatt
tgaatcgttggtggaaggaaatgacagtcccggaaagttatcacgtaggag**gtgagagtcttatctatcatatatgttct**
cagcctcaaatgcattaactcttacatagatacggaaaccagctatcgattaattttgatgacaacaaaattgtccgat
ctatccagctggtttatcattttcgttcgggtattcaaatatctcagtgtaccgaggtctaa*gagaagtggaagtctcgg*
gtcaaatcatttcaggagggcaaagattgttactacatatcagaaaactgactctcatcttgacttcaaatttatgaaaa
ccctcatttttaagactgatgaaataactacgggatggcttcaggattgatcgtgttggttacgtaaagagctatggat
tttacgaattagtctttctattgttcacggacactcttgttcatgtctatacacgatttacagctatagagtattttata
gtgtttttagtgttgtgcgaaaaaaaaaaaaaaaaaaaaaaaaaaaa

>*Bgh*Efc15
agacccctttcatcagaatcttcacattttcgagaaattccttaaatattcaaaatacacaagttgtccacaattgaaa
*taatattttaccgcagaa*atgaaaatctctatggttgcatcaattatagcatttctgagcaattttatgcctacattagc
tttggatggt**taccgatgt**gttgaacaaaatatttctcgtgaaatactcatgagagaagtcaatcaatcctatggcgagg
ctgtagagcaagatcctggcaacgctttttttagagacagcactttaagtgctaatcttgaattttctttcacattcccg
tcatataatgatccaaattcaacaataaaagtttttttcaacaatagaaaacaaattttacgagtacaaactttttttatt
tgggcaaattcatgactgtcatgaaattgctaaatggcatgcatag*caaaagattgatttacaagtatgaatcaaatatt*
tacacaaaaaatctatcaccttaacatggttaagtgttcatgatgcatggagataaattatacaaggtactggttgagtg
gactaggaaaagagcaacatttatgtagctt

>*Bgh*Efc16
agccacatggattgattcccatcagcctccctcgtctcagagtaggcaggaaactctaaactccctctatgttcaggatt
ctatcatttctaaacgaatcacaaggatatgaggagtcgaagtttctataagtagaattattctgttattgacagtctca
tacgataattatttatatcgacacactcctcagcaacctgtggtcaccaaacagctgaactccactcactttcactcagc
tcaaaaaagccgcgccatcagaaaaaacactctctacagcacatccaaggaaaaagtgggaaaaattgacgacaaatatt
*cgatcaagtccatatcatatccagcagatactgtattctcttcaaagattctaacctcaga*atgaagattactcttcttc
ctttcattatcgtattctccagcatttgggcgcctgcttttgcatcatac**tatgactgc**aacggaagtcatatttccata
agtaaagtacatcaagccattcacaattacgggaatttccaacaaaatcatggttatttattgaattcttttcgattaaa
acatacaaatactgccacaatcgattatatagatccgcgccatcaag**gttagcgaattccaaaaaaacccaggagtccat**
agctaatgacatttacttttatcaaaagcctatacattcttggttgattttgatcaaggggggggaattaaacgcgttta
ttctgcacttggaagggagatagacgaatgcaaatatatgtattga*gaaaccgaatcagaactagaattacaaaattgtt*
*gcgggcggtagcatgatgagacttaatgtaggtacatgcaaacgcctccgtataggagggacctg***gttagtagattggtt**
cttgtgctatggtattgcggctaaatctagatcag*tcatgacatgcgtc*

>*Bgh*Efc17
tccttgtgccaaaagatttaaaagataagaaggatgtgtaagattccgtaatccgcatgatatagctcactgcgatgcat
ccttctctctaattatttgttatatgtttcccagcccttctgaattctatcctacctctacacacgtcgtcttactattt
caaagccgacaactattaaccttaccaaagacgaaatttccaccaaaacagaggccttcgtctattgtaaccaggacttt
*cgactataacgatcaag*atgaagttctccaatattcctgagataacaacaattctgagtttatttacatcattatgcgca
actcaaggcgtgaatagtggaaatgcaaag**tacaattgt**agtggtgtaatattctactctgaaagtattaatgcttcaac
tgcaatggcatccacaataaatattggttcattgaatggatatcctgcaccataccccgtttatggcttatctggcactg
ctccgtaccatttatttcccatggtaaaagatgttatggtttacgcaggtg**gtaagttagaacacccttagaaattgaca**
tctttaattaaagctttccctaggagttgtgtcgaaattttcttaattattgacattaatgacattgagcagggtatgg
tatatgtaagagaaggttatgaaggctatttaccatgtactgccatgtaa*atggctttaattgcttgaataatggttctt*
cacatagtgtgataggctccttggggtaagatatttatctcaatcctgtcccaagtcatcaatacagtatcagaaaaatg
ggggcttctacagaagataagta

>*Bgh*Efc18
tacttgcttctgctattcatcccattccgttttcatcgcgcaagagtaacacctgtaagaattgacgcccacaagttatc
*ccgcaggcccagcagactacattcaccgagggtttattcaaagctccgactcaaa*atgaaaattgatctcgcctcactca
ccatcttctccagtcttttatgccagcactgggaatgataggc**tatcaatgt**gacagagatattatcccctcggaattg
gtggaaaacgctataaggggttcatataaatacttatatgaggagcagtatgacaaacttataaatttcgatgttgaaga
taaaattaatgtcgtactttcccatcagagtagatttggcgagg**gcatgtttattcctctctaattatacttgagtagct**

gctaatatatctatgcaaaaagactattctttttgggcctcacacaacaagtggggtgatttaattggagtgttttacag
agatggaacatttgatagggattgttatacagtgccatttaattaa*tgatacggagtgttatgataagggattgagattg*
ggatagccgaggggttcccagagaatattgaggtattggtacagaagaatgttggctggagagcatgtttcgacgctagt
gtatgtgttgataagatctgaggca

>*Bgh*Efc19
atcgagatttcaaaccaacattcttttcactcttgccatcataaatattggcgtagaacaacaacgaaaaaaatccacaa
tccaggctcatttgatcttgtgattcttctccaccgaaaccaggtctttgcatcagcagcttatattcccgagttcaaag
*ttgattccaagcatccgacactctcgaa*atgaagtttttcagctctgctacaaccgcggcgttggccggtctcttactac
tggtacctgccgcaaacggagaacaatat**tttaaatgt**cattctggaaggcagtttactatggaacgggttagaacatat
gcaaattatgctactactaggctatcacaaactattgaaccgactgtggacgaggcagaaacccgtgtttcatttgaatt
tgatcttctggattcgagaaatg**gtgagtctcccattcatctgacaattttcttattaaccatccttttctacaaccag**a
gaaatattattatttagttcaatttagcagcagtgaaagtacttattacgtgttcgagttgggtggcacatactggcagc
catgttctttccaccggaattaa*caggcgtcaaaattggtgctagtttaaaatggagagaatcctatatactgaatgtag*
agaggcttattcctttttgatagctaggctcatggttattcatacactggccagcctcacattatctgctataaacg

>*Bgh*Efc20
attcttcatcctttaaaaggacctgacctgctctctcgtcataagagcatcagttccctcacaccattcaagatattaag
cacaagttatcacgattaacttctttgacttatcaactcgagtcaccgtgacacttaaagagccttataacttactttac
*a*atgaaaaactttcttcatctcgctattgcgctcatgagcgttgtgatgccagcacttgctgtgaca**tttaactgt**tccg
ggtacaagattgatgagtctgttgtggcgagagaaatgaaaaaacggatcagacaaaacgatttatcgcaatccacactt
ggaagtcattcgactacttttacaaatacttcgatattactaccagtggtctaacttccagttcctctacaggttttcg
ttgctatactaacaccggtacaaactcacccgatgtgcgggaatgggacggagttagttggacaccgtgtagtcaggagt
ga*taagattgaggagatgcagtaactgcaatggctagtacttgcatggtgttaggaacaccgagaagatggaatcatagt*
ggcgagagtggacagagtgttacagtgtaattcacgaagggtactcagatagcttgtttagtactctgcctcagaataaa
ttat

>*Bgh*Efc21
atcgtcttctagtaatcttaagactacccagtgagccgttcatcccatcgccaatccagctcgtgcacgcattaactgtc
*tccttgtcccccccgaaatcccgaatcatcaaag*atgcgtacaaccactctcatcgcatgcgttggtacctggcttgtct
ctctggcttctacagtgcaatgtgcggat**tatcgttgt**gataatggcgctacaattccggcaagatatatcactagtgcc
ttggcaagagtcagagagcaagcgcaaaacggcgaagacggtttcaccctgcaacaaccgggcagaccctataaggccac
gcctacgactagatcgagcatacaattgtactggacccatatcgatgttgaccggaaccagtcttatgtgaacg**gtaagt**
cttcactgccttgtattctcttagccccctcttaactgaccttaatataggtgaatattatgggtacgaagtagtatata
gttctaattttgatttgtactacgtcaacgcatttaaagcaaccgatacgtggaaagaaaacttaattagctgtcaataa
acctgagagggccaagtgcagttataatctccactcaaattactgtaagagagagagaacattgtttactccttggtgaa
aa

>*Bgh*Efc22
agatatcatggaaataagactcgtgaagtctgtaagtataagaatattttacatgcggaaagaaatagtaacgaaatttc
actcttgcgatcataaatactggcgtagaacaacaacaaaaaaagtccacaatcaaggctcatttgatcttgtgattctt
ctccatcgaaaccaggtctttgcatcagcagcttgttgtcccgagctctaatttaattccaaacgtccgacaccctcgaa
atgaagtttttcagctctgctacaaccgcggcgatggccggtctcttactactgatacctgccgcgaacggatcttac**ta**
tgagtgtaatagtggtcaaatatttgatatttcaactgttcgaataaaggcagcatcggctactcatgagcaatcccata
ctattgaacctgatgttccctctcctcgggaacgtaagtcatttgcgttcgacgttgtaa**atgatcaatatggtgagtcc**
cccattcctccgatattggtctaaataatcattcttttctacaaccaggttcacaatggtatttagttcagtacaccggg
acattcccaacctactactttcttcgagttgggtggcacagactggagccatgtacattcaggcggagtaacagcgttga
aattggtgctggttgcaatgagacatcattgatatggaatgtagagaggctattcctatttggtacctag

>*Bgh*Efc23
aacttcatttaattccatcagtcacatatagagagataacacgtttgtttacccaaactgagtcgcaacgacaaatatac
agaggaacaactacattcccattgacaaagacgccttgttcactctagcaacgactactttgagtccagaaagcatattc
*aaa*atgaaattcctcttttcaaccttgactggcgtaattgttggtcttttaggatcgttgcatgtccaagcggctaca**ta**
tgaatgcccatttgggaggtactttacttcagacgatattctagccgcatcttcatctaggtcggcacccgacaaagtta
catatgcaaatggtcatgcctttaactggtacttcttgaaccgttatacacacaatgacg**gtaagatcacataggcttca**
aaaatttatcccagagctaacttctataccacaaagagtttgttgaagtacatgggctgttagacacaactacccgaaaa

gtggtacccatagcatcttttgccggccgcaattggagactttgttcgctaggccaaaggagtgatgcacaaaatgcgaa
tgatttgagcgactttgcaaacaaccactttgattactcatacatgactgcggaaattgaaaacgctttaagtgattagg
aactcctgcaacaatcctaaatttactagtattgaggctagctatgtttgatagcccgaaaaggtcaatttgttgaagct
ttgttgttgagtgatagagata

>*Bgh*Efc24

ccgcactctgacattaactcgtccacctgactcgtagggccgtccactcaactttt**gttagtccctccccatgcacttcaa**
ttgcaaatgccctcaatcatacgcttagctatcctcacaagctcaagagacagtcggactctaaccaataccaatacaga
agacatgactagtgctagctaaacaagatgatactgcaccgaatttaaaaatacccaatgcaaggaacttattgaaggaa
aatacacgatgtcttggctttgtcacaatcttccatgagcaaaagaattctagaacaacattttcaatcctttatcattt
tacctcataaaacggcaatccttgtgccagtaaattaaaagatttgtaagaatctgctattctctaatgaatcaattcca
gtcatatgaatccttacctctaatattttctcatgtatctccaacccgctctagagtatatcacacaatcactcaggccg
tctcgaactatttcaaagtctcaagattatcaattttaccagagaaacatttccccgccaacacggaagccttcatcttt
gataaccagaacgttttttaccttcgtgcaccatgaagttcctccatttcactgaaattacaacaattctaagcctatttg
catcactttgtgcgactcaggccgtgaatagtggctatccgatg**tataattgt**agaggtgtaacaatttactctgacagt
gttattaattcagctgcaagagcacacggattggaaattggagaaataaatggctacccaatggacatggatccttacgg
tgtaaatggccctcctccgcataaaagttttcctttggtacctgatcaagaggtttactctggtg**gtaagataaaatatc**
caatgaaattgacatctttaatcaaagttccatctagggattgagtctttttatttcgttattgtggacggaaatggcca
gcaccagggtgttgtatattcagctgtaggcggttacgtgccatgtcaagatcttgtgaatcccgagtattagccaaaga
tatccacgtgatatttattatgcacggacctttgtatacctctgctgctcagtaggcttggtagaagtaagacttttttga
tacatcaccattaaaatacaacacaaacacatttttagagaagatcagggtctattaccatatatagcatcatgagataac
tgtacgacagcttatttaaccctttgtcacggtctacgcataatcacttggggaacctaatggttaggggagacactaat
tacaatctatggtgaactacagattgaagaggatataaata

>*Bgh*Efc25

accattcgaagttccataatctatcagatctcacttgaagccaaccaatcagcaatcatgcgctatttcaacttggctat
catcctccaaagtgccgccttattcgtaccaacccttgctgactacaaatcccctcatataaatgaggggggagaaaagt**t**
tcaaatgtaacaaaaaaaattattaatgcaagtcaatactgggaggaaaaattgctcgcagataatctcatgcggcaaagg
caaaagataatagttcaagcgcaaggcttttacgacgtcttctggtcaagaagagaaacaaatttggtgttgtatgatga
caacactgcatcatactttttttgatcataagctcacgtcaatagcggacg**gtaagtgtcactgccttgtaaaatcaatgt**
attactgttaatttccaaggacagtgcgtaaatctcgaaagtttgagtttgaaatccggtatactctcgtgattgataat
tttggccgagtttgtgccatgttcatgaatgaacaagtagttggcgaagccgggtccagtttgaagcccg**agctcaaacc**
acgctataccaaatcattttgcaccatcaacagttga

>*Bgh*Efc26

Acctacacctgcctgaactactttgcacgtcggattttcaaaatgaaattttatcactcatcatttgtttcaaacttgac
aatactatctttgttcttgaacattaatggcgctttttcacgaacg**tggcaatgc**cgaagcggtgacatactttatgaag
aagatgtttattcacatacaaatatgaattcctttgaacactttccgtttaaaactatagaggatgaagtctatgaaaac
actagtttgagtcgcggtgtcgcttacaaggtactggatgtgcatccttcgtcatctagcg**gtatgtatctttgattatt**
tacttcttaacccctatatataaatgtgtttaattagacactaaaaacgtactagcaacaactttgctgtctttacttc
aaataaacgagtggcagtagtataccaacagggctatcgaaataaccgaaaattcattgaagaatgcacctaggcagaag
aggaagattgattccaaatactacgcccaatgagaatggtagaagaatgcatgatgtcacaagataccggccagaaagaa
agacctcaaagatacagctctcagctatttcaatttttctgggtgagaaagacgtcggccatgaaagtctgtatttaatt
tggagctttaacttgaaattattctccattggcgataaatctagctgtagatatggaatgcacttatatttagcca

>*Bgh*Efc27

aaggataagatgtgtaaatatccggaatcctccaaacaatcaagcctactagtattgatccttccctacaactattttct
acatattcctatctgctctagcattaatcttacctctactcacgccatcgtccgcaccatctaaaagttgacaatcatca
ccttcaccaaagagaaagcacgcaccgttgcagaagccttcgcctcttacatcaaggacccttctacctacaaccaagat
gaatctactccatttcactgaaatcgcgacaattctgagtttatttgcatcattaagtacggctgacgatctagaagacg
gttatgtgatg**tacaattgt**tttggtaaaaaattttactctgaaaatgttcttaattcagttacagaagcaatcaaacat
aaaagagctccaattcgtggctaccctcaaagctttaacgcagattgctacggtggacttcctccgtatactatattccc
agtcccagatattgaaaggtttgacataaaaa**gtaagttcaaatatccatacaaattgacaactttagttaaagtttcat**
ctagcttctggctatattttttgtagttatggacagtcgtaacgctaaagttggtatggcatataaaactacccatgg
tcacgaaccgtgtaaggacattttgttacaataacaaggtcatgaggggacagatgaatacctgaaatggatgcagatga
ttatggatgcgcggatatgattgatgtggc

>*Bgh*Efc28
*atctactcaaagttatcatctctcttctgacttcctatccacacaat*atgcgctccaatcgtttgctcaactctcttgga
atttggctcgtatctaccatagctgcagtgcagtgttttggtgac**tacagctgt**tccaatgatatggtaatttcggggag
agagattgacgcggcccatgataaggccaagtcgtacatcaataatgctgcttcatatagtagatacctaaatacaatcc
tgtacaacggaaatttaaatagcgaacctaattccgtgtattattggttcctggcacagttgaagaccgaccaaagaa**gt**
gagtctttcccaaaatacttacttctacttactaacgtactcaaaagatgtgacgactacgtactatttgctgacttcaa
cgactggcgaattttggggtaataagcactggcgactaccaggacattggaacatccgacacctggtgtagcccgtat
tag*tcggcaaagatagacaagtttccttcgactctgactacgcgtt*

>*Bgh*Efc29
gtcaaaggtaataaatagctgtaccaattctccccaagcccaaccatctactcaaagttatcatctctcttctgacttcc
*catccacacaat*atgcgctccaatcgtttgctcaactctcttggaatttggctcgtatctaccatagctgcagtgcagtg
ttttggtgac**tacagctgt**tccaatgatatggtaatttcggggagagagattgacgcggcccataataaggccaactcgt
acatcaataatgctgcttcacagaggatgtacctaagtaaaatcgtgtacaacggaaatttaaacagcgaatctgattcc
gtgtattattggttcataacacagtttaaggccgaccaaagaa**gtgagtcttccccaaaatacatacttctacctactaa**
cgtactcaaaagatgtgacgactaagtactatttgctgacttcaacgactggcgaattttggggtaataagcactggc
gactaccaggacattggaacatccgacacctggtgtagcccgtattag*tcggcaaagatagacaagtttccttcgactct*
gactacgcgtttaatttacagacagtgtgtcggctgtcgctggcaaaactaagcatagctcgcataattttcgggacttg
gc

>*Bgh*Efc30
cgcatcagggtcttggatctcgcaataaacaccaccgacattgttggtaaacacgcctttgactcttgccagcataaacg
ttggcacttaacaacaaaaaacgcaattccatcattaagatcaaacacatcttgtcttccctgtctattgtgctttgacc
*ttggattcgggcggttcgtcatctctagtccaaagattaggtcgacgggaacgaaaatcctcca*atgaagtttcttcgtg
ctgctacagctgcagcattgactggtcttttactcctggtgccagcctcatatggattaatgact**tatgaatgt**gtgagt
aagaatacaatccccatcgctaccattgaaaagtacgaaaaaaaagctactgtcgagaaagcgaaagaaggcgaccctga
agtccccagagggcagcggtgtaaagcaaaacgattcaccgttagattaccaaacagag**gttagtctgtcgcttcctaaa**
actttggtcttactaaccagtcctttctatcaccagaaacaatgttgtacttactccaagtcattggaccacaaccgtcg
taccaactgtacgaatatcatgaaccaaagtggcacccttgtcttctccgacagggcacatggctggcctcataa*cacgg*
ctgtgattggtatcaaggcaggatctggtagttgaatttgcacagaagctcttgtggatag

>*Bgh*Efc31
gcgcctttgagaaagaaaatatccaataatcaacctcgctgagacgtttatcattttcaaatccagccctctacactcca
*agaagctcagccgctaaaaaacaccgtttccacactccggcataagaggaaatttcccgatagtcaccgtcatc*atgaag
ttcttatactctgctacatctgcttctatactgggcctttttactacagctttttccgagaaaatt**ttctcctgc**gaatg
tggttatcaaatacttgaaagtgatgtaatcgccgcagccagaaatatcgatcttgacgcaaaaatatacaaacaagaag
caagtaatactgatagagatcagtccaggtttcagcttgatgccgttacatatgcggatg**gtctgtcgaccctcccctct**
acattaacactcagctgctaacacttagtatcaaggtttgtaccaaatctcagctataatttatatacaaagtatgacag
tccgcgtaattcaggttaaatatcgtactagacaggaaaaattctgcgatctcatcgaaggtcatgcctaa*taacttgaa*
gtgtaatcttctactacttaataagacccaaaaatttcgaaaacaaattagacggatgtcaggtgtaagcttatcttcag
attgttcagagatagataagcagtggttggtaagaacaactgaaatcgtgcacgactcagagtcagattgagcaggaatt
tattgcaactcatgctctatgattcaagtagttaggaattctacagccaaatttaaaatctcaggttcgaaaattcgaca
cctatcagtagtgttctctgcaatcctcaaactctcaccgtgtagataccaactgagccgatattgtgtcatagctagat
agccgcgtacgtaattcaaatcagtt

>*Bgh*Efc32
caagaagcatatcttgtcaatgatcgatgtacggtgtgccgtgttagcgccttaatgataccgagctccatcacctggga
ttgcgaattaaacagaaacagagggcaaaggacgagaccagatgatcgaggccaatacgtctccactgccagacctctga
atccgccggccgaaataagacagtgtggacttgatataaatcaagatgctccctccttcctatcgcctgcacctacgcat
caccttacctttccgctctcttggtcactttaactctaattctatcggtctcgtagggaaactacaaggcaacatcca
*cc*atgaagttcctctctataagtggcatggctttcatcctcagtctctctggctcggtcatgtcaatgccctggggccaa
gaagtcctcccggtacctgatccagtccggagcttcagc**tttacgtgt**ggcgggagccggacttatcataaggcagatct
tgatagatatgttgatcaagcgttgggcaggatgaatacaggtcaacccattacaacaacccaagcgttccaatatactc
acccaggaccatacttttcagcttatatcaatccggatttaaatgtctttagaactg**gttagatatttcccgacctattc**
tgcaggaattagccattcactaactatcttgatagaattcaacagagaagaacacgtcgtatttgatcaacatgggtata
ttatgggaggaatgacatatgaaatggttggaggcacgccaagatttgtgccttgcaaatttcctgcatcaactaaattc
atgtaa*ctgaagcgccattcatccaataatcatgcagtatggcccgaacatcctgaccaacggcgttgtcccaaagcatt*

gatgttggtattatggatacaaattcacgaatggattggggacagccaaggatgctctttgataaagccaggtgtgcaag
caagtggtctctacggggatagggggtttgcagtagttttgagggtaaaaagatgggtaattttatccagttacactt

>*Bgh*Efc33

aacattgtaaacgattcatcatccgtgaaaaggacttggccggctccctcgttgttagaacatctgttcctttacaccag
tcaagattaaaagcacaaattatcacgattaatttctctgacttatcaactcgagccaccgtgacacttgaagagcctcg
tgcgttgattcagaaaatagccctatctagatacaaccaacaactgtgaagagaacttacctgacaatgaaaaaatttct
tcttcatctcgctattgcgctcatgagcgttgtaacgccagcagttgctgtaatg**tatgattgt**tctggggacttgattg
acgggtctatcgtggaggcagaagagaaaagagtggcaagaggaagtacttctaaagtatgcttaattaatcctgattcg
acccaaacattccaatacttcgatatcaaaaccagtggttatcattccaatgaatatacaggttttcgctgctttatggg
caca

>*Bgh*Efc34

agtatttatcgtcttctagcaatcttaagactgcccagtgagccattcatcttatcgccaatccagctcgtgcacacatc
aactatctccttgtcccccccccgaaatcccgaatcatcaaagatgcgtacagccactctcatcgcatgcgttggtacct
ggcttctctctctggcttctacagtgcaatgtgcggat**tatatatgt**gataatgaagctgaagtcccggcaaaattcgtc
aatgatttcttaagtcacgccagagtgcaggcaagaaacagacaatctggttatagcttggaaaatccgggtgatgtgta
ttctggcggaagaaatccactgtattggcgcgctatcattgtttcgcaagatttagaaagattcaatg**gtaagtctctac**
tactatgcatgtttttggcttccactttattgacctgaatataggtcaacattatgaatacagaatagtttatgactccc
aatttaacctgcataaattggaagcagtcagactgaatggaggaggtagtgagacaacttttcattgttacagttattaa
aggcaacaagagtagttagaatctcagctcaacttaccgtgtgaggggaaaattgtttactacttgtcggcatattgac
tctaatcacgattgaagattcttgagtgtgtagctatctaaacctaatatagaaatataagcgagaggagaggctggaaa
ataaactagat

>*Bgh*Efc35

tacgattggatttatatcatccgacaagctccactgagtccaactgaccattaatcatgcgcttttcaagcatcgccgta
atctttcaatgcgcaaactcctttgggacggctcttgctgccttcaaggggtatcatattgatgaggggcttaaatcg**tt**
tgactgtgacggacttgttattagcgaacctgattatagagatcaacaatatagggcatcaagaagtattgaaaacaatg
tgcaccccccaccagactacgggagactttatgaatgcttatttagggaacttcagtgatggaagatgggtcatattttca
gatgaatcctcgaggaggttttatctttgtaatctcgacatacctggtacaa**gttagtcttttcgcacctccgcattgat**
aaactaaatctaactgtcacaaccagttgaaatagccgacaataaaagtcaagaaacctcctatgttcttgtactcgata
acctgggccgcgcagtagccatattcacgaagctcactgttactgacactgacatgtcgggtgaaagcttgaccggcaca
aaatatgagatttgtggcattgttcggtaacaagttgagtg

>*Bgh*Efc36

agacccctttcatcagaatcttcaccttttctagaaattccttaaatattcaaaatacataagttgtccacaattgaaa
taatatttcaccgcagaaatgaaaatctctctagttgcatcacttgtagcaattctgagcaattctatgccgacattagc
tctggatggt**tacaattgt**cttggacaagttttatctcatgatttactcacggaagaaatcaatctatcctacgacaaga
ctgtaccacaagatcccggaaacgaatttttacggacagcagtataagtgttcctctagtattttctatatcattcccg
caacctgattatccattgacaaaagtaaaaatttttattcaaccatagaaaacaaattatagctgtacaggcttttgcatg
ggacaaatattttaactgtgaagaaattattgaatgatatgcgtagcaaaagattaatttacaagtatgaatgaaatatt
tacacaaaaaagctatcacctcaacatggtaaagtgttcatgatgcatggagataaattatacaaagtactggttgagtg
gattaggaaaagag

>*Bgh*Efc37

Agtagtaaaagtcacacgaatatccaccaatatcaccttgatactcatcaaaagatccatcagcgcggaaaacagggcca
atttcaccagcgatatccaattcacccagtgacaaactttcaattgtctgtcgctcaaagtatcagatatttcattttct
cttgtgcatagtccagtttagtgcggaggacatcctcaaaatgaagtttcttgttacagcttctacggccgcattaagtt
gccttttttctcctagtgtctgctgcgcctggagagtatgtt**tatgcttgt**ggagattctgaccttttttacaataaaagaa
gctcaaaattacgcagtcgaagctactcctaatggaagatatgagagcgaccctgatagctcagggcatgacgtactccg
ggcacatcatttatgtagggttaataagtatgaag**gttagattaccttttttccgaaactatattgtcactaattattctt**
ctctaaaactagagtctggttactacgtagtccgagctgttaaagatcgattagcgctcgaattatggaaatatgttgat
ggaagatggatgccgtgccctatccttagcgtcgattcttgatgatgcagcatgaatggtctgcattaaggcgatatttg
gtattggaaattcgcgcaaaactcttgatgaaatagatgcataaaatgtttccgacataatttctagatgaaaatactat
cctccactgctcaaaactgctgacaatctcatgttctgtggctcgacatcaggcaaaaatcgactgacgtggttaacaat
catcttgagattggttgtctgtgtcagaaagtgctcggtattttgcaactactgcagctcttgaggagtacgctaccgta

acactcataggtcctggttctactagcgttagttttaaaggttctcttgcttttgtaacataatttccaggagtaccaga
catgaaggtctcggcacccaaaaaaaaaaaaaaaaaaaaaaaaaaaa

>*Bgh*Efc38
atcacccagattgtcgaccaaccttcctttgaatccattgacagaaaaattggctctgaatactgtccacacatatccat
cgaataaactttgatatcttataatttatcatacattaagcctctccaataacatctagcggcttgctttttttgagtcca
*gagtccagtcccgttagacgacattttcaa*atgaagtttcttagcgctgcttccacggccgcattttctggtcttctat
tgtcagcgtcagccttttttaatgaatccatac**tttgcttgc**agtaacgacctaaatatatatttatcagcgattgatcat
catgaacgacctgattttttattcggaggcgcaacccggtgatcctacggatgcgcaagggaattcatgcacggcataccg
acataccgcgagagtaaatggag**gttagtttcccttcctttaacctagccatcactaatgcgtcatgtctgtgaccag**tc
gatgttctcatcttgattcaattatcctatgaatttccctacaaccgagtctttgaaagaaccgagacaggttggcaaga
atgtccgtactacccatactaa*taacaagcaacaagtcggcgttgtggtttgtgttaaggcaagagctggtactagagcc*
*tc***gtgagtaattccctcacaacagaatgcaataaagaaaaaagcctgctaatactgttttctcccgttccag***gaccagct*
attctttcaacgcagtcttcacgcctctcagtacctcccggatcgacgcaccccgtaaagtcggggacaaataccgagag
cttagctctacttgagtagtgcatatccatgatgggaatttcaatgcgacattaccaagtagtctaggcaggatagtttt
atttgtgttttgtttattatcgtggtaggcaatttgcacctcccccactccaac

>*Bgh*Efc39
*atattcaagcaacaccaactacttcatca***gtaagttttcattaaatccatttaggtcaatctggttctaatcaaatgaaa**
ag*aaagttgtaaaaaatcaaactcccaggttttgaagataaaattactagggtcatattcaaa*atgttatctcgtctttc
tttgctgtccattttgtttggacttttcatctctgaaagcgccgcaagt**tttgcttgt**ccaagtggcaatatattccaag
acaatgaagtagagccaagagcaaatgaaatatacagtcagggtattaacctagatactgaacgaggtcaacgatcaaat
tatcaaggcatcacgttccttggtagcagaaacagtgattattttgctttcgaagctcccttttctcgtcctgatatat**g**
taagttaaataccctttcctaataatatgacagcttctaattcttctccccataaaaagcggatagaacatacaaaatta
atgtgcaatatccatcacgacgctttgatttgatagagatgacacaacgatcgagtacaaagttagaaagtacttgtgat
aattattatggctatgaagtataa*gctacacttaaataactgtaccttcat*

>*Bgh*Efc40
agttaccaacttattactgcttctttgctctcattaattatctatcagatagaaacaagtcattatttttttacaacaaca
*gctgtgctagtgcttcaat***gtgagttactcctgctgtttatttttcctccacaacccaccaagtaattcatatgggagaa**
gctgaaggcaaaatag*atcagaaaaccaaattttcccaagttgaataagctttgcataactcaaga*atgaaaatccagaa
tatatcttgcctcttgacaatcttaactacatcaagattagcatgtgcctacgaggtt**ttctactgt**gacaatactgcaa
ttacggacaaccatgtacgaatagcctatagcaatgctcataacgcacctacagctggatatccaagatcttattggaat
gactatatagccgaaaataccagcctccaaatatatcctattttccttaatggtgaaacaactatta**gtaataacatatc**
tttaattttgattttgaaaatatatgctaacttttcaagatacatctgatcaggtatttttagcctggtttgggcaagga
gtaagttggggagttgtgcgaatatctggttcgggtgctcaattatgtgatagagttgatcgtggaacgtctctagaact
gagtactatagttacttga*tagaggatagcaaagttgatattggaaatggggataaatatctga*

>*Bgh*Efc41
agggacaagttttccgcggcttaaatatagccagctttcacaaaagcgaatcagataatcacaggactattcaggagcac
*caccaaa*atgagaccattccaacttctgtctgctcttgcaatctttattaatctggaggcagtagaggctgcagcttat**t**
gggattgtgatggtactgaaattccagaaagaaacgttcgagctgcagtagtacttgcctttaattatcgaaaagagtct
tttcatggttatcccgcaacttttattataggatctacattttctggagtaggggaagtacgacagtttccagtagaaga
ttctgacgcaaactggcaaggaggagctgtaaaatattatatttgacaaacaagagggggtcttatctcgaagtttttt
catcagttggatctggcaataagtgtacttttgttgagggctag*gcttggcttttttgttgctttagggctcattcttatc*
tcttgagcacatgagagtttctaaatctactcag

>*Bgh*Efc42
taagtcgccacacaattccaacaacagttaactttttttataccccttctcattgtaaaatcagaacctcaggaaatcaaca
*aggtccataaactaggcttccacccaaaaacatacaaacaacagggtacactcagg*atgatattcaaagattatgctttg
attgtcgcaaccctgattctatgtccgaaagttttggctagttcgttg**tatagctgc**aacggagtagaatttaccgagga
ctacgtatcagaaactgtggagcaagcagtcaagagatacattcctcaggcaagacaacattctcttgctgataatgacg
cgatttacatgtactatccactccttacaagtggacaactctatcataaaggactgaaacctgaaatttattttgttcgg
attgagatagcaacaaagaatttccaagtagtcatgatgaaaaatctagattatcctccgggggccagtgtaaatgccaa
tccgcctttgtgtgattacattgaaaatcataatcagtag*tggaggggtagctggatccctggcctaagacaaccgaagt*
aaacttcaagggacccgcatagaccagtcgaaaaaaagtgattcttgactgtgatggtctatcaggcttcttcaatgatg
gaattaggttctcctcttacgccagcttcgctaatattttgtcgaacatgtccgaatctattcaacactcttagtgctat

cctcacgacaaactcgcaaatcgatggactttgttaacggtcttttccttcaatgaatctaagctctacttccacagggg
tgatagatttcatgaatatttggcgggattttaaatggacgcggaactattcaagaaacctcgcaaagtatcatgggtta
ccaagagaatttacaactacaaagtgaaattgaatcttagtacaggctgacggcaatctggagcttct

>*Bgh*Efc43
*atcatcatcattatcatcatctcttcaattactttatta*gtaagttttaattccatatatctgtatgaatctagtcatat
attgaagtaccagaaagattttaaccaatagaaattctag*aaag*atgttcaaaatgagatctgctcatattgcttcatt
gtcttttattttggcttcttgattctggaaagtgttgcagaa**tataaatgc**ccaagtcaagttcgaataccggatagtg
atgtcgagacaagagcaaatgagatttactcaagaggagtatacctcgacagtaatcggactcctggtgaaaatcaaatt
gaagaaattgagttctatggtgattcaggtagtggagatttggcttttacaggcgacttctctcctcctttctcaa**gtaa**
gttgatttcatcaccatggttataaaacagctactaatatttctatttatctaaagcttctaatacatataaaattacgg
tcgagtattcaccaaaaaaaataatactaacagagaaaaatacatttgtgggtggaaacattgaagcagtttgtaaaaaa
tattaa*aacttaatattattagcaatatttgtcgataaaaggataatctttgggcggtatgaattagttctctaaaactt*
agaga

>*Bgh*Efc44
*atctactcaaagttatcatctctcttctgacttcccatccacacaac*atgcactccaatcgcttgctcagctgtctcgga
atttggttgatatctatcataggtctggtgcagtgcattacagaa**tattactgt**ggcaacaatgtggtgatatcacagga
actaattcaatctgtcagcgaaatagctatctcggaattaagggctggagttacatggcacacatccttatccggtatca
tatatagaggaaatctatgtggtttaccaaacccgacctatcgttggttcatacaacagtcggaagatgagaattcgg**gt**
aagtgttagccgagcaagccactttgattttcatttgttaatactgtcacaagaggtggtgatgaaatactttttactgg
tctcggccagttgcgaagtgcagggggtcataagcacaggctggaggaagaaacttggagaagatgacacctggtgtacc
gaggcataa*aca*

>*Bgh*Efc45
agtataagaagtctgtgagatagccataacaactctatagctcaacaactcgcagaatctttggcttctttactaaatca
tccttcaatatcctccacctcactcagttgttccccttattgagttgatacatcgctcataataagagaggataatatag
tgacattcttgactgttgctgaatcttacataccattcagttccaaagacatctacgaggcacctgttacaataaaccaa
*tatcacagtcaag*atgaaacttttaacccttgcttctacaattgcagcattcagccttttgaaccaagtagatgctaatg
cagaa**tatcgctgt**cagggacatactttttactgccgattatatacataccgtagcagcctatgcctggagagttgagctt
aacactcatggcagctttccccaagtggcacaacttgcccctcaatctagtggatatgggcctgccagacagtttccact
cctcacttcaggccaaatttggcaag**gtatttatttttcttatctcttatctcttatcatcaaatagctaatcactttct**
aataggtggaagctgcaattatgtggttaattcaaatcgggataggactgtgttagaagtatacgaatttattaataata
gctgggaattttgcagagc**cacaaacctttaa**

>*Bgh*Efc46
tggaatatttgggaatacaattatctttcttcctgccatatctctcacccaaatagttaagcgccactcctttgaccttc
atcgacagaaacatctgcgttgaatagtataaaataaagcattcattccaatcacatccggctctgtcttgcccaagccc
*tcgatcttgcaatcagacagctcactgaacccagcacccaatctaatctcgtgacaaggcgccgtcgga*atgaagttttc
aaatactgcctcgatggcagcactggctggtctttcgctcctgatacccgcggtgcatggaacattaatg**tttgattgt**g
ggaatgatgccagatttcttacttcgacagttatggaatacctatcaatagctacggccgctagcatagaagacgatgac
cctccttatgctcaaggacaagtcaatggcgcatatcgatttactaaaaacataaataatg**gtttgtttttcccccacttt**
ccacttcgctctcactaaccattgatgtctgcgaccagttaatagaactttcttagttcaaagtactaatatacatccat
tttaccgattttcgaaattacatctggacattatgaatgtcctctcaaagaacactatgcttag*caacagtgacggtgc*
tatgaatagtatcagggcaggatttatcgtggccagaaaccaccggaattcgccctccctgaatctgtctacacgcacct
tgttgcggtgcaaataggacatgatgatagtcaagacttgatgtgaagtatttgactgttgtttaagcggggatgggcat
gtgggactttgcgtgattattatgatttttttatatatatcaatgaatgtgcaacctgctatctagctctgacacatgac
tgatgctgcgtctgcgggttggatgactcctttgacgttagaggta

>*Bgh*Efc47
acaattactcaggccgtctcgaaccatttcaaagtctcaggattataaaatttaccagagatagatatcaccgccaacac
*ggaagccttcatctttgataacgaggacgtttttaccttcatgcacc*atgaagctcctccatttcactgaaattacagca
attctaagcttattcgtgtcgctatgtgcgactcagggcattgatgatggctatgagatg**tttaattgt**agaggtgtaaa
attttactctcaaagtattactaattcagctacacgagcacacaatgctagacttggatctataaatacctacccaaagg
tatatcatggcacagctggtggtcctggtccgcatagaatatttcccatggtgcgtgatgaagaggtttactctggtg**gt**
aagataaaatgtccattgaaattaacataattaatcaaagttccttctagggcctttgtctttttatttcttaattgtgg
acgcacaaggccagcagcagggtgttgtacaaacattggcaggcggtcacatgccatgtgaagcatattag*agaagatct*

gggtctattaccgcatacaacattatgagataaccgtacgacggcttattcaactctctgtcacggtctacgcataatta
cttggggaacctaatggttagaggagat

>*Bgh*Efc48
atcagagtatcaccttccgctcttcagatcgctcagatatcgcacgtgcacaatcaaagctatatttccaagttgacgct
*gtaaacactttattttgag***gtatctgaacagtcagagagacttttgacacggctaaaatatacacag***aattttcaga*a
tgaaattaattcacattatttcatccgttgcaatttttgtcttccggcattggcagttaatgat**tgggattgt**gaacaa
gcttatatttctgcagatgcactagaggcattgatgaatagcgtcgcccgaaatcgtgaagaaagagaacgaggctcgtt
ttttggtgcttcatcagaacattatttcacagtcttgcgcaatggcgatgacttagattatgcac**gtgcgtaaattctat**
cttgttaagtctttcacagctactaataaattcatctatgattaggagctaaatttgaaatatcatttaacaacgaattt
aaagtcggccaattgttaatgaatattgatgacgtcataactagatgcgttgatacaaataccaggagatag*tttattgc*
tcctgttggattacttttgcaagtaagaatgtgaaatcgggaacgggctctttg

>*Bgh*Efc49
cacttaaactactttaattacttcaccgatgagctttgccaaaaaaacacttgaatggatttgggtctaagtcaaaatac
*aaaacaaagttctaatcaatctgaatcatcattcctgaaataagaattgctagcaatatattaaaa*atgagatctccaca
tcttgtttatttgtccgtaatttttggatttttcatattcgaaattgctgcagtt**tacaagtgc**cctagtcaacaatata
ttgatgactttactatcaatacagcagcaaataatctttacgaaaagggtttacagtttaattttggccggcatcctgga
caatcagaatgtggtggtatcatattttctggtagtacagctaatcacgatcttacctttacgagagcctttagacctcc
atttacaa**gtgagcttaattcctgacccttgtaatataatagctactgatattcttctccaccaaaag**ctgttatgtcat
ataaactacaagtttctcatccatcaaagcagattacattaatagagtgcgggataactgaagaaggatatacagaaaag
gcttgtcaaaaacagtag

>*Bgh*Efc50
cgtcgacattttgacaaattcatctttgactattgccgacataaaagttgcagaagaacatcgaaaaaaggttaataat
cgagactcatagctgttacttccattctactaagctttgaacttgatatcagacggctcagcgtcccgagttcgcatttc
*gattccttgaaaccacattcccgaa*atgaagtttatcagcactgcgacaaccgcagcattggccggtatcctactactgg
caccagccacttatggaaaccgatat**tacaaatgt**gaacttagcgatcccgtgagcgaggcagatgttataaaaatggtg
tctgatcgtactttggaaaaaaacgcagatatacacccaaaagttccaaccggggaatcgcacaagtcttatatgtttat
gaaaagaa**cgtatgcaatagttagttatcccgctcctccgaaattagtcaaattaaccgtttcttacaggaccacgtat**
aactgcgtacttggtacaagtttatggggaccctaaagaatatcaactttctctacatgccggagggagatggcatttat
gttctctggaagatgagagttag

>*Bgh*Efc51
tacgcgtgaagcatatttcacaacgcacatgcgaatcgctttaatgcgaatattttatttaaccttgtcatgacacgtta
ctacaacatgctttacagttagacggcactaccaatagatctgttcaggcatccagaggaaggctagatgattgaaagat
gaagaatccccacaaatctagatttagcatctgctcagatttaattggcatggacatccacaaagatggctgagccagat
aaataggaagttatcgaccatgtctctgatgatggagagtatccttctacatcacccgacagacctcactggagaccaac
*cgatcaatcatc*atgcgcttttctaatctagcaataatcatatacgctgctagatttatatctgcagcttccaattacaa
aacggcccacataggagaggaaagaaaaggc**tttatttgc**aacggagaatacttctacgaagctcagtatagggaagtta
gaacccaagcgctaaaggttatgaaaaatagcgatatgactgcctttccggcgggggagcttgagaaaattttgaggct
gaacagccagagaggttgatgatgtatgataatggtgacgcaaaagggcactatttattcttgattcccagtctgacgag
ag**gtaattttcagtctccatcaacaccgaggcttcacggttaatcaaaaaatacag**ttcaaacccgaagatcttacagcc
tcgaatcccagtttaatttcctactggacgattctgggcgggtgtgcgctttcatggtgtggaagaa**aaagacagccaca**
aggggcacgagtaccgaagaaccgacgtacgaggtttgcagtatctcatcgtag

>*Bgh*Efc52
*atcgcaagcatacacttccaccacgtaaaagattttgctggaggctaattgaaaat*catgcgtatctccagcatagccac
aatcattcaagtcgcaagcgtcttcgtgacaactctggctggccacgctatgactaattataatgaggttaatgga**tttc**
aatgtgaagatcgctatattagtcaggggaaatttcaaacgcgcgactatctatgacaagggtagcggaacatgaaatc
ttcaggccattaaatgatagactcaacgaggcaatgagagatccaaatgacaatagatacctcatgtttcagaatgatta
cgccggtcactacgagttttacttcctaagctcaattgaacagg**gttagtatcaccgctaccttgcatcgacatttcatg**
gctgaccgtttatagtgttcagtaactcagatcagcagatcattaagtacaattacattctcgtcgttgacgattatggt
cactcaactgcgatgttcaggagagaaacagtccacacaactgtatcatcgcaaggatggttgtatcctgacaataccat
attatgcaccatttattag*tggaatcctcaaca*

>*Bgh*Efc53

cggatcagtgtgattcgaatacctttgaggctttttagggaccctcatacttatattctcatcatattccacctaaatt
*catcctcaaaccccatcattcaactcaatcatcccattcatacatcgccggctttgcgcaatcaacg*atgatgcactta
ttcatgacaggcgtattcctgaaagtaatcttcgtcattgtacacttgtccacgctctctgaagggcgaggt**ttccagtg**
cagcgcaggagtaattgttgatcaagacctcgaatccatccgtcagctcgcttgcaacagtctgcaaacaggcaatagaa
agtcttcagtcgacaataattcccagggcaacgaagaggttggctacgagtggaagctgcccaaacctttggctttggaa
caagttaatg**gtaacccttactctgccaaatgcacactgtctcggctgcaaagtttaattctaattactcctag**gtggaa
tgattacgcaactgcagctcaccgagaagtgcgaaatcacgtccatcactgtgtccagcaaagaaaatgaaagccaagtt
tgtccaaaagaaatggctatccgtaaacgtcaaaatggaacagtgatatgccagggtgttttattccgcagtcaaggtct
gattgattccgccgctcgttactgttcatatgggcgtgttaggagagacgacgttcttggttttccaccagattacaccc
cagccgtatttagtactgtaaatcactacatggcagaagtaccttattatgataaagtcaatgactatg**gtaagaagcct**
cgtcgttctagaaatctcacctttgactaaatttctatctagactatgataaattaacgtatgtggtctttgatgaaagt
tgccgtattgtgggagccatggtctttgcagattataaatttcggtcttgtgagtttgagtacaggggttga*tttgattt*
catttagcccgttcaggtaccctatgggttcatgcaggaatatctagcta

>*Bgh*Efc54
atcatgccatatatataagaaagactattcccacttccattccagtatccagttcacaaacctcgccatcgcttccaata
taacttgaactacttcagactcttacaaagcacctactattcctttcttcttcaaagccgatcaacctacaagatcaaaa
*ataccaacaaatccagtcactctaccaaaatataagcaatattcattatacaaaatagatacaat*atgaaaattttcagt
cttatttcattggcggctattcttagtcatctgacgcccgcaattgccatttcatct**tacaagtgt**aaggacaaagtcat
tggtccgattcccctacaacaacttatcgacgaagaatttagaaggcaaaataagacagttggtccaggaccggcggatg
aagtttgtgttacaataaattttatcctcaccctaaagcatcaaaaaa**gtatgaagtttattccaaaaaacattatttcc**
tactaatttatgtcttcagatcacgtcaatgtttactttactattgaagcgaacaaacaaaaggaaattgttaacttaac
ggcttatgttaataaccagaatcctgtatgtgaaccaacaacagaggaacctcattttaatacattactggatatataa*c*
cagcaccatgaattataaagacaaaaaatctgatcgtagtaaagtatgtcacg

>*Bgh*Efc55
atccacaccatcaaaatccacccatgtcatttgagtagcagaggtcgcataccagccgattcaaatacacttgcattgaa
*aagagaagattcaac*atgaaaccgatcaaccatgcttcaattcttgcaatcctcggctttttccaagcctccggcgcctt
gtcc**tttgactgc**aatggcgtgcagttcacggatgactatgtatcgcggtccgtcgactgggccacgacgaaattcgaag
ctcccctagatgaaaagcaccggctggaattcgatgcacgctacttctactacccactccttgcttccggggaaatctac
aagttcg**gtgcgtagcccatctccaccgccctcgacgcctgctaatgcgcacaaag**ggatggcgcgtgagaatcattatg
ttcgggttgagagggtgagccgagctcggcaggtagttagagtcagcgatgccagtcaaccgccgaatagtcgcgtcaac
gagaatccgatgctgtgcagtagcgtggactag*tgtcagt*

>*Bgh*Efc56
agagtccgtcacattctaaacatcgaacaaatagtcaactaatacgcatttgacttcgacgacagagtactggcacagag
ttttataaccacgatctcataataaccacctagaaatttagtcatcaatcatctattggacttagatgataaaagaagta
*gcattgtgtttaagtacaaatttaggatacgttaatacgacattttccac*atgaagtttttcagctctgcctcgacggcc
gccttgacctgccttctgtcacttgtgcccgttgcgcatggtgaaccatat**tttaaatgt**actcaaaacacaatattcac
catggcagaactccaacgtgataaagtatatttttttagagacaaacgcgaagcacggggatcctttaggtccaaacggcg
aagcttaccctacacatcgatattcgatgataccaaatagggtt**gtaagttatctgttcctctgatattgctgccacta**
atcattcttcttggtgaatagacaccacaacatatctatttcaattggttgatgaagctcctacattccgcgtctttgag
aaacagagtgg**agaattctatccatgcaatttgatagaaaattaa**

>*Bgh*Efc57
cattcaaaagacgacaccatcctcatttttcttaattcctaggcaccgagtcaacctctaacggcacattgagccatcg
cgatcctgccattaagtatttatcgttgtactttcttgagagtgtccaaacagttgagaattctccgccatcttcagact
*ctacgacccggccgct*atgaagctttacctttcaaagttattaatactcatcttttatctggtcgggcccgcaatagccg
ttatgaga**tatgaatgt**agaagacattatttcaccagcgacgctgtaaaaggcgcagttagtcgagcaatcagatttggg
tcacatttggagaaaaccaaaactctgtttggagaaccacgatacaaggtctttattgatctcatatttgatggatctg**g**
tttgtcaatataatacccacaatcatattacttactgaaattaataattgaaaaggtagcgctgctttcgcaataatcac
acgaagaagccataaagttaaaagtgtgatggttcgtg**atgaagaaggtcaatgggaaacatgcagttatggtttcacct**
tttgtacaaacttggatagagctggatggacatacataccaggggcaacaggccctggttgtagctgcggagacatgggg
tga

>*Bgh*Efc58

attgcaaatgtatttatagatttcatggaagtcaacctcgtcaacttatgagggtaagagagtcttagtcctggaagtaa
agtccattatcaaaactcattacaccttatacttcctctccatcgaacctcgatcttcatattagacggcttactgtccc
*gatttcacggttcgaccccgacaaaattacattctcgaa*atgaagtttttcagtgttgctacagtggtggctttggccgg
tcttttaccactattacctaccgtgtacggagagggatat**tacaaatgt**cctagtaccaagacattcggagaatcagata
ttatggaatacgaacatctgccttctcctgataacaacgactcttcacatcctgaagttcccgcagggcaaacttgcagc
tcatatttattcagtggtgactcatttgatatag**gttattttccccattcctctgatatttttctcattaatcgttcctt**
tctacaactaggtatgaagtttatttatttcaaat**aattgggaaaactccaaaataccgattatttgaatttacttctg**
atagctggacgaaatgtcgttttagaaagcagaattga

>*Bgh*Efc59
atcccacgacatattggcacctataagaccgacagtgaatctcctccggtactaattcttcaagtgataatatttacaca
actgtaaataatttctcagcatatttcttcactgacacacccataaattcaattcgtcacgacttgagggcccaagaaag
*agctacgcaacacttttcttcgacctgaccgaaagccttgttgggggcactttcaag*atgaagtttttctgtctgacttt
cgtgtcgacgttctccagcttattagtttcagtaaatgctggatcgttt**tatcgatgc**ccaagtaaaagttacatcttct
ttgagcttgtcatgggaagagcacgagaaatttattacaaattgaatgcggaagggccacaatacaacctggccaaaac
atgctcggtggaactgctatttggggtagtatggaggatggagacttattttatgaagtccccttcgttcctgacttaa
aa**gtaagtaaatatccaactatttaatacggcggctgttaatctctttcaattataag**ccacaaataaatataagatctc
tat**aaacgctgcttctacagaaatatatttaagggagactacacaagattcaacaggagattgtagcacaggaccacctg**
aagtccatccaccatcgaataaagtgactgattggttgtggaaaagatattag

>*Bgh*Efc60
aacttcatttaattctatcagccacatatagagagataacacgtttgtttacccaaaactgagtcgcaacgacaagttta
ctgaggaacaactagattcccattgacaaagacgccttgttcactctagcaacgactactttgagtccagaaagcatatt
*caaa*atgaaattcctcttttcaaccttgactggcgtaattgttggtcttttaggatcgttgcatgtccaagcggctacat
ttgaatgcccatttgggaggtactttacttcagacgatattctagccgcatcttcatctaggtcggcacccgacaaagtg
acatatcaaaatggccataccattaactggtacttcttgaaccgttatacacacaatgacgagtttgttgaagtgcatgg
gctgattgacataactaccaaaaaagaggcacccatagcatcctttgccggccgcagttggagactttgttcacgaggcc
aacggagtgctgcacaaaattctaatgatttgagagactttgcaaacagccactttgattactcatacatgactgaggac
attcgaaaagctttaagtgattag*gaactcctgcatcaaccctaaatttattagtgttgaggctagctatgtttgattgc*
ccgaaaaggtcaatgtgttgaagctttgttgttgagtgatagagataaga

>*Bgh*Efc61
*cggagaatcgaaataccatcaatcgacaagcttcattgagtctaacaatc*atgcactttttcttacctggcgctcatcctt
caaagcacaagtttcttcgtgacgattcgtgccgctcacacgacgagtcacattgatgagcaactgaaggaa**tttcattg**
tggtgaatacttattgaagcaagaaaattatgcagatttacaaaaatcggaaataacggcaatggagattccaatcttct
ggaatttgagtgccggagtcagaagagcgatgggagacacggatgataagagaatcgttttgtatggtgacagcaagctc
gatgagctcaaatttttcaatttgaaaacattattgagaa**gtatgattacttttgcacttcaaacgttcccagctgaaaa**
tgcatattagactacgaggaaagtggtaaaaatatattcaagggctactatctcattctcgatcaaagtagtcgtgtgag
tggtataatagagaggacgtttgaaactaacctcagaccgaatttgaaaaacccaccacgagaaactttctgcatgtgca
gaatt**gatgtgtaa**

>*Bgh*Efc62
ctcaagaggaatgaataataagtgtattctgaatctacgaacgagtcatttgatttctgtgctatatatatccagcagt
*ctccagcagtctccaaaaatcagtactcttcggcgccagattctgtcaactaacagccacagctcattcgacg*atgaagc
tcttcaccgccagcggcgttgcgacactcttcagcattctagcgcctgtgacagctgtgtcagttccgaatagtggaagg
ggaataacagtgccaaacccagatagaaatttctca**ttttgtgt**gagaatgggcgcagctacgcaaaagattatctcat
aggaaaagtcgaggaggcgataaatattatgagacacccaaatacacattatgattatccagtacaattaaatcaccttc
attaccacatcctcggaccattgtggtattatcctgtcaatt**gtaatgaaagaatccatgaaatttagatgccttccgaa**
taacttgacctttactgactgttacactcttctcatagtgcctgctccagctgattttgtcgtgttcaacacgaactctc
gtatagcgggcgtcgccacctacgtggatcttccttatggcgaaaggacattcagagcctgctcactcaattaa**gtaatt**
ctcccaaaattaattgccgtcttttcctcttcattcatattttattcagctttatatctacgacacagctttatacactc
aacctctaaataatattcacacaaatatctttcacattatccttgatttctttctaaacattaaatttatggattggaat
tgtttttgttcattctcggatcatcccttcatgtcctcctgacacgtattgatatgactaacagaagcaagtag*gagtt*
gaatcgaaccagcgttgtcgaagccgatatctttatcaattgaaagtcacatgatacaaggtcgaaaaatattccggat
ctccaaagtcagatgatcatccctgacacgcaacacacactcggcaaggctgatggagtcggtcgatcccatcgagtttc
*cgaactggatcaatggctatt***gtgagctttgtacccataaagttgtgatgtcttgttgacgaaaaaaag***ctatattcaca*

atgaaatcgccctatctacaacatgctatctgtcgggaggtagtgacaatgttctttgctgccaaggatcgtaaaataaa
cttgtttaattcttgagatgaacactcatctccg

>*Bgh*Efc63
atcagatagtcaactaacactcatttgacttcgtcgacagaaagctggcacagagatttatcaccacactctcataatca
ccaagaaatctcgttatcaatcacctattggacttcggtgataacagaaccggcatttttgtttaagtaaaaatttaggct
*acattagtacctcgttcccaac*atgaagttttcagctctgcctccacggccgcactggctttcctcctgtcacttgtgc
ccgttgcgcttggtgattcatat**tttcaatgt**actcataatcaaaatttcaacttggaacaactcagaagtggtaaatca
ttgtttaaaaagtcgaaagtgatgcccggcgatcctctaggtcctcgcggcgaatattatcgcacaaatcgaacaacctt
gatacttccggagggtgt**taagttatcttttcctctgaaattgctaccactaaccatttttcttggtgaatagtccaggt**
atcatatatatttcaattaattggtggacctcaaacattccgcgtctatgagtattcaggtggagtattcaagccatgcc
ctttcatagaagattaa

>*Bgh*Efc64
aagccatttcgattcctgacttgatctctattaaccttcaaacttcggcatttcctagcttgagatttggaagcaacacc
*accacgaaacactaca*atgaagttttcgccatcaccggcgtttccacgctcttctgcgtcttagcgccagtgacagcga
tgtcacgtctaggaagaacagcggcatcaccagtgcctgatccgaaccaagaattcagc**ttctcgtgc**ccaaatagtaga
gtatacgaaaaaaatcatctcatacaagtagttcaaacagccagacaattgatggatcaaaatgatacagacaatggtta
tccctctacattcaatcagctttcttacgacattaccggagcattgtggcatcatccacttgaaggggcctaggtggac
aagatttcgttatattcaacaccgacaatgtaatagtgggcgtagccacccgcaatgtttttaatgaccgagtagtcttc
cgatcttgtcaaatcacttaa*gactttgagagggtgaatgatgt*

>*Bgh*Efc65
acagacactaccatcgtcctcactacccccagccccatctatccgccacccgcccgac
atgaagtttctttctgcagtcgtcggcgtactgctctacagctcctccattcctgtccaggccatgtactcacccgcaca
aagtaataccatccaaccagtaccagtgcaggactacaatttaacc**ttcacatgt**ggcacgaccttgtacactaacggac
ccatcggtcgaacgttagggagagtctacaaaactagaagagaatccaaaccctatccaatggaattcaacgcaatacag
tatagggtatctaccaccgtctacatctttccccttctgcccagctttagattatacagcgatg**gtctgcgtgctcctca**
tacttgacctcagacctcctgccggctaacagctcgcgatagaaaaaaatccggaacacttcctcgttttcaacgatgac
aaactgatcttgggcggggtttatctgatggagacgagcgagggtcgcgtcttcaggccgtgctactttcccatgaaaac
acaattcttgacgacttga*actagccctaatagtgtaatgctggcaacggaagctcgg*

>*Bgh*Efc66
cattcaaaccactttaattacttcacctatgagctttgccaaaaaaacaactgagtggatttgggtctaagtcaaaatac
*aaaacaaagctctaatcaatctgaatcatcgtccctgaaataaaaattgctagagatatatcaaaa*atgagatttccaca
tcttgttcatttgtccgtaattttggctttttcactttcgaaagtgctgcatat**tacacctgc**ccgagtggtatattaa
tccaaaacgatacagtcaggagcagagcacaagaaattatgaatctgggtgtgcagctagatgttcatcggacacctgga
caagttgaaatgtcaaatattacattttatggaagtaaagcaggcggggatctgtggtttactagtgactttaatcctcc
aattacta**gtaagttaatttcctcactttaataatacaacggctgctaatattcttctccttcaaaag**ctgaaaacacat
ttaaaatttcgatttcttatccaggagaggaaattgttttaacagaattccaaaaatttggttctggaagtcaagaagga
acttgtgatgtatctgttgaaatgtag*acatatcagactgaactgcacgcagtgcggagtagacgaagactactcatctt*
ctaccgctaccagctttggtgaggtctcggttggtaatgatctcagtggtgtgaatgttattccatcacgtaatcagaac
ataatgtttctacctaagcgaggtaaaacatagatagacaattatgatatgatatgattattattatcgtaatcgggtac
tactccagcacccagagggcatggcacgggtaattcagatctatatgactcttcgattactacat

>*Bgh*Efc67
actaccattcataccatgtccctgtcttattttacactttacttccaacaagcctacataccatagtaaattgccaaagg
aacttcgtgttttattgcggatgggaaaggtaggagtgatggggcaaatggagataaatcttgttcacacataagcactt
accatttactcatgcgtataaaagggatcattccaacctcagcacctatatcaagataacaatcttcaaggcagctttca
*gtacaattcgaaatatag***gtacttataactctaacactaccattgcgatttcctgtcatgaataaatccgacctttcgag**
attgaacatattcatctagctaattcaccaagtacag*atatacagatccaaaatttataagcataacctacaac*atgaa
aaacctaagctttgtttcgcttgcgcttttcttaagccatttgatgcctgtattggctgttaaagat**tatacatgt**ggcg
gtgtagttattcctgcaaaaaagatcagaaaagagattaaaaaaaaatactacagtctgccaatacaatatgaccatcaa
tactcggcaacggg**cgaacgtgatagagtttcatttgactttgtcccgccagcaaaggtttgcaggaatcgtgccaatt**
atttgttatctcctacttacacttaaaagcaggaagttcaaagttatcagttgacataggatatgatatgagactagaga
tcacgtcgatgacagttactcgggatagccaggttattgattgttactag

>*Bgh*Efc68
actacctgataagctacatcattcaagttgaatccaccttcatcttacaatcttcgcgtcgttattcagctgtccaaagc
*agaattatcgtggccgtgctcgtctgaataagaataatacctaattcgagaaattttcaca*atgcagttctttcgttca
gccttagttgtatcgctttcgatgctttcagtctcagccatcatcctcgatggtgta**tataggtgc**cctggtcttggata
tgacttggacgcgaaagaagtggcacaaaaagtcgacagtattaatattaatgcagagaaggaagattggtatgacttcg
aagaaaatggcgtgcagtataaaaatatttatcattctgttgttgaaaatagtgaag**gtccgtagattttctccaaacac**
ttttatagatcttcctgctcagattatccagtcgcgaaacatattttcataaaggcgaacaagaataaagatgtggtagc
ggttggtgttcatcaaacctcggagatgatacaatgccagcacataccagcagaggtacatagtaataaaacctcagcac
tcaaataa*ttgtctcgaaaaaatcggcgagaagctgaacatgaagaaatatctcatttttcgttccgataaaaatggaac*
ttaaattgcaaacagtacaaaattatcgcatcattacccactttacgtttcgttcgtctgaggtaggtataagcccaacg
atggacgatgaatgtgttgcttgatgggattattcatgcttaggagtgagtcatttattagtcatcgctctttcgatctt
tgagccaagatgctacctcattattcaagaagactctactacatacttcacttagtttgacgcacaagatgtcctgagta
ctggctaaggcggaagaattcttcttcgcgatgaccgggagtcggctttagttcactgttagaaaatatagcactagttg
aagctggcatttagttacttagtcaaagcccagccaagctcaggtatttttttggtttgctctacatgaagttttgaatc
acaagcttcacactttagattatgaaatataatctgcttactttattccgaaaataatgaccaccgagtcttaagaaca
gatcataatcgcctacgcaactat

>*Bgh*Efc69
*acgtccaattgattgttcgttgcatccggaactttctcgagtctag***gtaagcgcagctcatgcattctcttcttcctctc**
tacctcacactagacatcataaacgggcacagaggctcatggaacatgcaataagatctcgcacataagagcaccgtcta
cacgcttgcaaaatgaagattcaaaacatgaagtcgttcatagcgcttatgtttacaccaaatttgcatatgcagatta
tgtc**ttcaattgt**tatggtttggatgtaacacgagagcttatccggcgtgcctatttactttcgatgaatggatatgtgc
cagactatcccgtaattacatcaatttcatatctatacaacaatgaaccgatcggcatctttcccatccttggcgatgac
gaaacgtcgatagcag**gtaactttcttccacctttgattaagctttctgacttggacaaag**ctgaactgtatcgaccttt
cttaatttggagcctatcttttctagattggggtgtgtattttctgggtgataacggctat**gagatctgttacaagaacc**
cagcttataacgggtatacttttagacagatagcgtttgggaatctatag

>*Bgh*Efc70
aggtctactttggtttgtgaatatcctctacactttgcactttcattcaccaattacctctaaccagactgaaataaatc
*gaaaactattatc*atgcgcttgtataccattgcactccttcttcagagtgtcagcttctctgtggcagatcttgctaatt
atcagctacctgatattcaagacagccaaaaaaagt**ttcaattgt**ctaggccaagtttttggctaccgtggtttgaaagat
tatattagcaaggccaagggttatataagtcaaggcttgttgtatgaaattacgattccagaaattgaagcaagactcgg
gaatttgccgcacgaaagcagagtgatgtataaaagttccgagccggatagcaaatactatcaactttcaatacagggtg
atggagagg**gtaagttattccaatcattaagagagccctatatgttgctaataagtatccaaag**gcagcagtttttacac
ttacgta**cttattctcgactcacatggccatttatctgccatttgtggaaggttaccacagtccaatctgttgcaacag**
gcattagcgtaattgaaaattttgtaccatatcacaatga

>*Bgh*Efc71
acatcttctctattgaaaactcaaggctacccattgagccattcatcctatctctcatctagttcgtgcacgcgtcgacc
*gctcacttttcccttcacatccgaatcttcaaac*atgcgcacatccactttactgagatgccttttttacttggcttgtca
ctgctataacgacagtacaatgtcaaaatgac**tatcattgt**tccaataatattgtggttaagggcgacgttattcagaga
gctctcaatgatatctcaactgactcaacgtcgaccctctacgcggcaggtacattatatgttaacgacgtatttgtgaa
tcgttggtggaaggaaatgatagtcccggaaagttattacgtaggag**gtgagagtcttatctatcatatatgttctcagc**
ctcaactccattaactcatacataggtacggataccagctatcgattaatttatgatgacaacaaatttgtccgatctat
ccagctggtttatcattttcgtttgggtattcgaatatctcagtgtaccgaggtcatagaggaataa*gagaagtggaagt*
ctcgggtcaactgattacaggagggcaaagattgttactacatatcagaaaactgactctcatcttgacttcaagtgtat
gaaaatctccattttataagactga

>*Bgh*Efc72
agttaccaacttattactgcttctttgatctcattaattatctatctgatagaaacaagtaattatttttacaattaca
*gctgtgctagtgcttcaat*t**gtgagttacttctgctgtgtatttttcctctataacccaccaagtaattcacatgggagaa**
gctgaaggcaaaatag*atcagaaaaccaaaatttcccaagttgaataagctttgcacaactcaaga*atgaaaatccaaa
atatatcttgcctcttgacaatcttaactacatcaagattagcatgtgcctacaacgtt**ttctactgt**ggcaatactgca
attacagacgaacatgtacgaacagcctataataatgctctaaactcagctatacctggatatccaagatcttataagaa
tgacgatatagcctacaataccactctcaaaatatatcctcttttccttaatggtgaaacaaggatta**gtaagaacatat**
ctttaattttgattttgaaaacatatgctaacttttcaagattcctcctatcagtgctatttagcatgggttgggcaaga

aattgcttggggagtagtgataatctctgatatgggtactcaatcatgtgaaagagttgatttcgaaccattttacaac
tgagtaccatagtttttggataa

>*Bgh*Efc73

atagctcaataactcgcagaatctttggcttctttaccaaatcatccttgaatatcctccgcctcactcagtagtacccc
ttattaagttgatacatcgctcataataagagaggataatatagtgacatccttgactgttgctgaatcttacataccat
ccagttccacagacgtctacgaggcatctgttacagtaacccaatatcacagtcaagatgaaacttttcaccgttgcctc
tacaattgcagcattcagccttttgaaccaagtagatgctaatgcagaatatcgctgtcctggaaatacttttactgccc
attatatacataccgtagcagcctatgcctggagaattgagctttacactcatggcgaatatccccatgtggcacaactt
gcccctcaatctagtgaacatgggcctgccagacagtttccacttctccttgcatcccaaatttggcaaggtattttatt
ttcttatctcttatcatcaaatagctaatcactttctaatagcggaagttaccatcatgtggttatttcaaatcaggct
aggactgtattagaagtatacgaatggaggaataatacatgggaattttgcagaggcacgaatatttaa

>*Bgh*Efc74

agtctcatctttccctattcttcacatcgttcaaatattgcacaatcacaatcaaagttctttgccaaagctgacgctgg
aaactcaaatttttgaggtacctaaaccctgagagaaatttatgacgctgctaatacttgccaggcattatccaaaaaa
ttaaattctacaatgaaattaattcacattatttcatctgttgtaattttagtctttcggtattagcgaccaatgattg
ggattgtcgaggaaatacaatttatgcatacacaatagatagagagatgtattggtgcttccataaatatagccttacaa
atccctcggattatataaatattccagaacatcatattttcaaaactgagttcaaagttcccggatcgaacccgtcaggt
ctgtaaattctgccttgaagagtctttcatatctgctaatgtgtctgtcaatgttaaggagctagatttgagtttagatt
taataaagactatgtaatcagctatttcgtttataaatataacggtcaaatctttgattgcaatcgactaacaacttcaa
caatggcctcagactaa

>*Bgh*Efc75

acccagaacacatttgtatacttttctatttttactttctctctccatcaccagactcttggcagcagcatctgaatctt
acgcaaaaaatccaatcaaagcccaaaaatccaatctacaaggtaagtcaacaaagttgccaagctttaaaatattcaag
taattaaggttgcaccaaaatgaagtttattactcttgcatcattgacagcagttttgtgcataacttttcctggaaatg
ttatgggagattacaattgtaatggccgacacattccaatttccctactacaggaaaatgtcaattcagcctgtgcaagt
tttgctgcagtaaatccaaattttcttaatacaaaccccgaaaacaaaatttacactcataaattcttatataaagttcc
ggaaaccataagtaggtaaatttcatatcaaaatgattttgatagctgagaattaaattccaccagatgaagtatatata
atagtgttttatttcaataaaagaaagcaaataacatatgtggaagctaccttccaaggaataactgtgagttgtctcag
aatctcatagtaatctaagtggcctaggataggctcttgtactggaagtgagttgcataggctgagcaaaggcacttat

>*Bgh*Efc76

actggcaacacattatcccatcattcattggttcaagctcacttttaattcaacgaattctatcctaccatcaccagcct
ctcaaacggaacaactccttcactcgagacatctcactgctaaaagaaaattgaatatcaatttgccaaagatactttca
aaatgaagctcctacatcaggcttccattattgcaatttgtgccatctcgttacagtttctgccacgcgacactatgtt
tgtccacaaggaaagacgtttgaagaggaatacattgagggattgcgggacgacgtaatctataggcgacagacactttt
gagagtcattggtgttcatgatacccgtccactctatgaaatcgaaaatgtgactgtaggatcgggtcagtttattcttt
cccgtgtagcactacctgctgatttttcttccatttcaagtgacgcactacttcgtgttatcgatagatctttccactat
ggcattccaagtgatggaaaaagccggagataactatcttgagtgtgatctacggtaaaactagggaagtag

>*Bgh*Efc77

agacgtattggttggtagagttttctggcaacaatatccctcgatatctgggcacctacaagaccgacagaaaatctccc
ccaaactagatattcaagccataatatttacaccactataaatatttcctaggctttttttcttcactgaaaaacccatac
attcaattcgtcacgacttgagggcccaagaaagagctactcaatactgttgttcgacctgacttaaagtcttgtcggag
gcactttcaagatgaagttattctgtctgactttcatgtcagtgctcctcagcttctcagcttcagtaaatgcagcgata
ttttatcgatgcccaagtagtattttaatctcgactaagtatcccttggctagagcacaagaaattcttcagaaagtgaa
aatgatggcgttccagggaccactcggccaaaaaacggtaaatggcatcactatttccggtagtatggagggcggagatt
taatatatactggcgagttcgttcctaactttaaaagtaagttaatcccctacttttttaaatcggcagctgttaatttat
ttcaattataagccgaaaataaatataagatctctataaacggtattactacagacatgtacttaacggagactacacca
aattcaacagaagagtgtacaataagatcggctgaaggcagttcacaaccaatcagagtgcatacatggttttaa

>*Bgh*Efc78

tctctctcatcctatgtccccttcagaatatcatttctcgccaatctgcaaatagcctcgacccgcatgcaatttcttaa
atcgttcccaaataagaatacccattaagtatcttagagacaaggtcgtctcaagtacaagtttccgggactcaaatat
agccagctttcacaaaatagaatcagacaatcacaggaatcttcaggagcaccaccaaaatgagaccattccaacttctg

cctgctattacaatcttcattagtttggagatattaggggttgcaggttattgggattgtaatggtaccctaattttata
tgaaaacgttcgagctgcggtagatttgcctttagttgctcaccaggctcttttatgagtacccctcaatttatcgtt
cacaacctccatggcctggtctagggcaactacgagagtttccaataacagcttccgggaaactatggcaaggctcgttt
ttcagttctctttagtaatgaaaattcgctaataatattgataggagagtatatagcatactatatttagcaacccaa
gatacgagttattgccaagtctttgcaacagaaggacttggctatgaatgtactcatattttgggctag

>*Bgh*Efc79

agagtctcatctttctctattcttcagatcgttcaaatattgcacaatcacaatcaaagttctttgtcaaagctgacgct
gaaaacacagatttttgaggcattatccaaaaaattaaattctacaatgaaattaattcacattgtttcatctgttgta
atttttagtctttcggtattagcgaccaatgattgggattgtcgaggaaatacagtttatgcatacataatagatagaga
gacggctgacaccttcactaaccattgcactggacttgacaccaattattgcgaaattaatgcaggggatattttcgaag
ctaagttccaaattgccggatcgaaccctccaggagctagagttgaagtgaattttaacaa

>*Bgh*Efc80

tgagactccaaccatctaatatccgacagtttcctctgagtctaatccatcattaatcatgcgcttttccagcatagcca
taatccttcaaagcgccagtgtctttgtgacaattcttgccatccagacaacgaggcacatagacgaggacaaaaaaaga
tttgattgcaacgtcggcctgctcgagacatatgggattaggacaacgtattccgtgcctcaatcacagagacttatgcg
tgcattatacccacttcaaaatgcaaaactcgatgaattgctacaaatgccgaacgatcaaaggacagtgttgtattcag
atggcctttctccaatttacaggtttcatgaaatgaattatttctcggagcgtaagcattactgcactttcacatccaca
aattagagctgacattctatgctagacaagacatccggtgctaaaaccattgaccgagaccatattgaagtagtcgatca
aaacgatcgtcagtgtgcagtggtgttgagggaggtaactcgtaaccttgagtcgcaggggtcggtggaagatgtagtaa
cttttgtattctgtaacattaaataa

>*Bgh*Efc81

atcattcaaattgtcgggcacctgttcctttgactcttgctgacagagatattagcatcgaataataaaaactataatcc
atttattctcatcacatcatgtttttccttccccatgtcctttgttttgcaattagtcagctcactgtctccagcactca
aatttaatctctcgagatgaaggttctacttgctgcttcgacagcaggcttggctgctttttgctcctggtgcctacag
catatgggctgcataatttcaaatgtaatagtcgaagaatcttcgcatattcagttattgttggatacacagattcagct
acctacgaacaaattcaggccggggaccctcattttgctggtggacatacctacggggcatatcgatttacagattatct
accagatggttgttagtctccccattccttcaacttagctctcactaatcacgattttcaatgactagctccgagaaatt
acttagtccaattggtcaatgtgaacccatactaccgattatttgaatcttctggaggccgatggcatatttgtactatg
gaggggagctagtttaggtagaataatgtcgctttaatttgaagccaaggggatttgtgaatggaatgttgcacacga
tcattttgcagcgcaattgaattgagaatccaatttcataacatggtcagtgaacaccgctctcttctttgaccagaccg
actagattgactacgagagcaaaggctgactacctgtcagccatgtactcaacaaacctggcagttttgaaaatgtaga
gtacagagttacgtcaggtgatagttactggcaattaatgataaaggatttgttagattttctactagttctgtggtgg
gatttacttatcaggaaactagggatcctgtcctgtagcaggtgagaagaatttacggatatcaacaactcattataaat
aacttgaatcttcct

>*Bgh*Efc82

agcatctttctacttaacaatattattcctcaacattgaataccaaaaacttcaaaaattacctaaggatactatcaata
tgaaattatttcaagctactgctactattgctgtagttagtaacatggttacttgcgtgactgcagtgtattggcaatgc
cttagtgtatcatttcgtgagcctgttgttgtcgaagcagcaacttctgcttggagcaatggtttaaattctgtcagcaa
ttaccctaaaacttgcttctgggaggatccctcgggaattgatgaaatctttcgatcatccccaatgaacgctaatggat
taccttggacatcaggaccagctaactactatgttgtgagcaacagtgatagaagtagaattaaggtttttcgaactta
gatggtggttatcaatgcaatcttattcagtaagacatggaaaaataccaggctgaaattatttaaactatgagcag

>*Bgh*Efc83

tcagcaaagataataataacttctagacatcaaaaacacatcttcaagagatcggcttcaacatgaagacttttattccg
aaactcgccattgtgctattcagccttgtagtcccagcactggcagatggctggacatgtgccggcagtgacttcgacgg
tgaccaggtgagggggcaagctagacattgggttgatcagcagaaaacaaaatttccgactttcaaacccatgacgggg
tacaattatgccatttcgaactgggcgaaggaaatcaacttactcaatgtaagtggacatcaattgcatcaaactcccac
aatgctgattcttgtatgtcaaaaagcatttacagggttccgcgcttactgtgatgaacccggtgatgtatacaatgtta
gatattgggatgttagttccagaacatgggtattgtgcacccattatgacattgattttgaaagcgattaccgttag

>*Bgh*Efc84

atctgacatcagcttcctcggcctttcacacttcttgagggcactacaggttccaattaccactgccaccatcccattcc
tacaaaaccggtcattggtaatagtagaatcatagtgtctgtcagcaaagataataataacttttagacataaaaaacaa

*atattaaagagatcggccacaac*atgaagacttttgttccgaaactcgccattgcgctattaagccttgtagtcccagca
ctggcagatggc**tggacatgt**gccggcaggaactacgaaggtgtcaatgtgagggcgcaagccagacattgggttggaga
gggaaacttaaaggttccgatttag**agccagtaacaggagcaaattacacttttttcgaagtcggcgaaggacttatac**
ttactgaatgtaagtgcatatccattgaatcaaactcccacaatgctgatctcttgtgtgtcacaaaaagcatgtacagg
ggtccgcgcttactgcactttgaccggtga

>*Bgh*Efc85
acctatcctctcgtctgattcacctctgccaacaagtcattctcggcgtgtcgagctaacttcaagcccagttgctaaag
*tccacaatttcgggccgattcaataaaagttcatcttcttttaatcaggacttgagtctatttcccttcagg*atgaagat
ccagagttttacaagattaatggtattcctgggtttgtcgctatcaggatgtgctgctgctcct**tatgactgc**gatggaa
ttcatatcaccatgaatgatgtttcgagaacttataacttagtcaattctcgaaaatacgaatcacagctatttataatc
agtttaggtaaacttttacagctataa*aactgtccttctgtttccaatgtcatttgaagacagtagtcaagctgcacaag*
ggggagaattttatttagtcatgactttgaataggagcgagtgtggtgtagttcaaaaaattggttcagagtacaaaaaa
tgttcataataacca

>*Bgh*Efc86
acataaataatgttggtatccgacgcattgttaggactacagcgactttgtcacaagacgttggctccatccccatcatc
atgcggctcacatctgtgatcgttattgttgcctttctttcagctcaagtattcgcacaatacaaagtagccaggaaaca
gaac**tacgactgt**ggagtaaagttgagattaaaatcatctcaaatcgacacggctatgacgcggatggaacaagtctata
gaagctcatcgccatcatgcaacaaaaaaccaactctcttgaataaatgctgcaaaatttgtgtaagatatttaacgtat
cggggaactaatttcctcctccggtaaatgacgaaatattgctccaaaatgtcatcagatttagtttactccgtaaaaa
gcttttga**gtaagtagttcttgcatgttcctcttggtgtattttgaaaattacacgaatactgactgttgtag**atcgata
ttatgtcgttgctcgatgttcacatacacacggctgttcgtttttgggcatcatccgcaggcctctctacaaattgagag
agattaagtgtagtttgatacagaaagataaacccgcttccagggctctagtaagctttccccaacctgttgcaccgcca
ggctctgcaccaccttattcccgtcactga*tcgcaggccgttgaaagatg*

>*Bgh*Efc87
aagactgactcttttgactcctcttgacctaaagttggtgtaggaaaactacaaacatactttccacaacatccacatac
accttgaaatttgttatccagcagacctcggcaataatactgagccactatcccctcccagcctcagcttttctccttgc
*agaataaattctcaaa*atgaagtttttaagcactcctttaactgtagcaccggctggctttctcctgcttgccccagctg
tgtatggagaaaattat**tatgagtgt**ctaaatgaccaaaaattctctcagcagaccattatgaacaggggggagatcggct
acaattgatggagcgcgatatggcgaccccaaaggacccaatgaggagacgtgcaaggcatcacgattctcaatcacaac
ggcatcgggag**gtgagtcttcttcctacagctaagacgccgctaacaatttctctttgcaactag**tttccaccacctacc
tagttcaagcaattgataatccttcatcctaccgtgtgtacgagcttcaagattttcaatggatagaatgtcattta**cga**
cagggatctacaaatagaggcagatggtga

>*Bgh*Efc88
cggcacaaacacttcttttcggagaagcttctcccattattacttaaccaaagccgcaccactatcacagacattctca
tctcgcagctcacaaacctccagactgaacgaaagcattgaccaggagagctcattttaatagcaaaggaatcattctcg
*gccaaagtcatttcgaa*atgagacttctaccgctggcttcattcatcgctttattcggccatctcatgacagtgtccgc
gtacaacgaa**tataaatgt**ctctcgggagtccggttctataagcatcaaattcatgaattaaagacccaaatactcgaac
acagaaacgggcatcaattaacattcgttgaacttgacggtgatatgccagtctatgagtttgaaaatcaaaatattaat
ggcg**gtcagtctacaacttccataatgattgacgaaagctgctaaaatatccacctctgaaag**tcaagcacttgtaccag
ataaaggtaaatctcaccacggagaatttatgggtgatggaggtgatcaatggagtccgtcattac**tgtaatccaactta**
g

>*Bgh*Efc89
aatcaagacttatcataactattgttacctctctactcatatttgaattcaacatcagaaaacttagcttctttagttta
*aaattcaattcagcagaaatctactattgaa*atgaagttgatcagctctacttcgaccatggcgttggctggtcttttac
tactgataccagcctcttacggaagtaaacaa**tacagatgt**cctcgtggtgatcccataagtaaagaagatgttttacga
tattccaagggtcatagtgagagttttaacctggatgagtaccctcttactcccgcggggaaaaaatactcatcaacata
tttcacaagtccacttggtgcacctc**gttagtttggccattactccaacatctgccttttttaactgtttctttctag**taa
cgtatgccgctgcctacgtaatacaagtgtatgataagcctccgcaataccaattggctcaatataacatgggtgtatgg
actgtatgtactgtggaagagagtcaatag*ccactgaaattccgcagtcatctgcatcaatgtaggacctgctaatggaa*
acttgcc

>*Bgh*Efc90

attgttaatcaaattcatttagagctcaagtgtcacttcacatacgtctaagccaaagttacattacttgaaaattagca
*gtaacgcttcaaatctcagtccaagaag*atgtattcaaaactcgctatagtcagtgctgtcattgcaattgctcaattgg
cagctgctgggtct**tacacgtgt**cgtgacggacaatcctttactgaaggatatgtgaacactatcgccgcctattgtttt
gatatggatgaagaatcctatgccggctacccaaaaaaatacgaaaacgagcagctaggtcctagttgcgatcaattccg
catgtatcctcttgttccaggcggttctgaatggggtg**gtaggagctcaatccttcttcatgtattgaaacttaccaatt**
tttaggcggatcttttcaatattttgtcgcaagtgacagtggtcggcaaacaaaaagggtttatc**agaggagccgcagat**
ttgacgaggattgcccatatacaaggtag

>*Bgh*Efc91
aagcccaacagcataatacatacccatttttcattggaatagtttctctctatttctcccatcgtctacccttacatactc
gtagctggagtttcttccattgttaggctgaattcgctcagagccggtaaacttgccgaccaaaagcacgtcagtccat
*tcttgag*atgaagcaacagattatttattggctcattccgctcctttatctctcaacagtagagtccaagattgaagac**t**
acacatgtttacaagtaaagataaaagcgaaatatgttgaaaaagctcttgatttttttgaagaatccagacaaccagatg
tatagaatgcccatgactttttggacatgtgaatcggatcattattgctatatctctgcgcttcatacagacgatacatc
atttgagaggag**tgggcaaagattctaaccaaaccggctttccatactaatcatttgcgcagg**atccacgactccttatt
atgtggttgtagattgtaataatgattttagaggcgttgtagtgagcataggcacaggttatcaccaatgcact**ccaaaa**
agacccgctgttatcattagagcatcaaattag

>*Bgh*Efc92
*gagcatctttctacttaacaatattattcctcaacattgaatac***ttggtccctcctctctttgtcctctaaattattcta**
gaatgattcttacttatttaattattgatcacagtattgatcacagtattgtctcaactcgaaaacttatctaaaacctt
attgctgactgcttatgcatatttaga*caaaaacttcaaaaattacctaaggatactatcaat*atgaaattatttcaagc
tactgctactattgctgtagttagtaacatggttacttgtgtgactgcagtgtat**tggcaatgc**cttgatgtaaagttta
gttggggtgttgttgtcgacgcagcaaattctgcttggagctttcgtctaaattctgtcagcaattaccctaaagcttgc
ttctggggagattcctcgggagatgatcaagaatttcgatcattcccaattaactctaatacaatgccttggacat**gtta**
gttgctgaagtaccttttatatctataaaatctctaatatcatttatagcaggaccagtttactactatgttgttagtaa
cagtgata**gaagtagaattaaggttttgcggatttaaatggtggttttgaatgcaatcttattcagtaa**

>*Bgh*Efc93
*aggattgaagcttccatcaccccatacatttcatcgagtctgaacgatccataatc*atgcgcctcaccagtctagccgtc
atcctgcaaagtgcgagtttctttgtgacaatattcgctgtcggcactaagagccacataaatgaggaagataaagaa**tt**
tgattgcagcggtcgcaaattcttgcctgcagaatacaatcatgtggagagaatgcaattaactgatccagttaacgaac
taggcttgactatggatggtatatacaaagacttattgcaagacagaacagaaaagggcatgtgtgccttcaaagacaaa
gatgatggttactataaatatttccagctcataaatttatggacaa**gtataatgactccagtgaattgagtaatcccatc**
taacaattatctacaggccgcaaagtggaaaatggtaacgctatgtatagttatatactcgtctctgatcgtaataaccg
t**gcgaatgccatgataaagaggatgacgatttacgctgtcgatggcacctcaaaagaatcatactccatatgcaaaatca**
gataa

>*Bgh*Efc94
agaggacgtggagacaatgatgaaatgaagcagctcggcccatccgaacatgggagatgacgagaaccatcggcaccgaa
gaccacataaaggatactcgaagtagataatctagtcatctaaacatatcgaccatcaatcattcaacgacgagaaatcc
*tatattcatcaactgataaatttcctctacgtcttttccaaacaca*atgcgcttttccagcacagccatcatctttcaat
gcgcaagcatctttgtcacaattgtgattggaagctctacagatcacataaatgagcaaagtaaatca**tttgactgt**gat
ggaatcattgttgggcacgaacaactttcacagaaacgagacttgatacaaagaccattgatcggaagtaaaccaatgac
tttttctaaaatatacccagagaaat**attatgagatagtatatctctatcaatcacccaagaagtatgttaagtttggat**
atgatgatcccgcgcggttatctgtgttccgactacctaaaatgttacagtgtaagattcaaatcttcccaacgtcaaaa
tag

>*Bgh*Efc95
acaactcatcatattggctacttgttgactgtttgtgggagtccaactctcatcaaccaataaatctactggagaataac
*caataatc*atgcgcctctctcgtattgccatgatctttcaaagcgcgagcatctacaccacaagccttgccgctcgttat
gcagcgcatcctcaagagaattataagatt**tttttttgt**gatgttgaatttgagcaagagatgtatgcgcggatagagcg
tgagaaaataactgatacaggtactattcagactttgaatcaagaactcaatcgtttagttgcagacactacacaccaga
ggtttgttcagttctatgacacagacaacggtggctacg**agtatttcaattttccaacacgatctcgtagtatgactatt**
ctgtgcattccgtcatcttatctaactatcgtgaatagatag

>*Bgh*Efc96

agtccaacttccatcaatcagcaaatctactcgagaataatcaatcatc
atgcgcttttctcgtatggccatcatctttcatagcgcaagcatctgcacaacaagccttgccgcccaatcttctagaca
tattaatgagaaatcgaaggtatttcattgtaatagtgatatcaatcaaaggcaatattcacggacaccacattataaaa
tacaagatccagataccattcagggattgaacgaggagctcagttatttagttgcagacacttatgatacgaggactgtt
cagttctatgaccaagacaacggtgactacgagtatttcaatctttcagaaatatgtgatagtatgactgttctgtgaat
tttgtgatagtatctaacaattgtgaataggatttcaagagcaagatggttctgttgttattgaacacatactcgtcaac
gaccggcaaggtcgtgcatgtgccatgatgatgcttacaactgtaataccattaaccaattgggacggacaaagtccaca
aagatattatagcttgtgcgcagttggctcgggataa

>*Bgh*Efc97
tgtaagaaagactattcacacttccattctgtatccagttcacaaacctcgccatcgcttccaatataacttgaactact
tcagacccttacaacgcacctacaattcctttcttattcaagaccgatcaacctacgagataaaaaataccaacaaatcc
agtcactctaccaagatataagcaatatttattatacaaaatagatacaatatgaaatctttcagtcttatttcattggc
agctattcttagtcatctgacgcccgcaattgccatttcatctttcaagtgtggcgaatctgtcattggtccggctacca
tacaagatgttatcaataacgcatttgctactaaccaacagtcacttgttccaggatcggatgataaagtttctcttaaa
acatattttgacgtccctgtaatgcttaaatcaagtacgaagttttattccaaaaaacattatatcctactaatttattt
cttcagatcaagtcattgtcagatttgaactgggagcgaacagtagaagggaagctgtttacttaatagccactgttaat
ggccagaagattccatgtactccaacaacagagaaacctgattataatgaagttctggcaagatcttaa

>*Bgh*Efc98
aaacagataggcaaataacactcatttgacttcgtcgacagaaaaactggcacggagttttatcaccacaatctcatcat
aatcatcaagaaatttcgtcatcaatcacctattggacttcgttgataaaagaagcgacattgcgtttgaagtacaaatt
taggatactttagtacgtcattccaaacatgaaattttcagctctgcctccacggccgcactggcttgtcttctgtcac
ttgtgcctgttgtgcttggggtaccatattttctatgtcgtgacgaccaaaagttcacgctggattatatccaaagtttt
aaatcaaattgttaggtgagtgtagccagcgagggcgatccgctagatcccaacggcaattttaccccactgcaagact
cac

>*Bgh*Efc99
agcgttgctatcatccgacagacctcatcgagcctaactgatcagtaaatatgcggttttctagcatagccataatcttt
caaagcgcaaacatcttcctgacagcccttgctgtctacaccacgggccacatatctgaggagagtaaaatatttcgttg
tcgccaacgcgatattactgagagagatttgcaagcacacaacggaaagaaataactcatgtggcaaatgataaaattt
cttggtcttggacttcccaactaagtgacttgatgacggattccaggaatcagaatgttgtcttgtttggaaagaagtct
gaggtttccaagttttacatcctcgaaagattgacaacaagtattatccctccatggtattgtcacggcaggactaacaa
tcatcaccaggtgggacttctaatggttataatcatacatatgaatatatcataa

>*Bgh*Efc100
accctttcaaccagttttgctcaagaattacattcacataaacttccccctcttttcgctaccattaggacccaactat
agatttcgacttagttctcgcaacagggtagtgagcatttgcggcttcaaactttcattcagcccttgattatgtccagc
ttgaatagaaagatagagatacggctgttatggttattattcccagcccagctcatcagaagttcgcaaacgccagtgga
gtcggaaatcagactatttgtatgcgtagctcgttcagaattgatgaaacgccaatccaatgatggaaagttcatcggat
taatttctcttgtaacccgccgcaactattgagaaaattctatgccgcatttcttgctcgaaactcgtcattagtt

>*Bgh*Efc101
acagcaatcgtcacgccaacgttccacgtcctccacgtcggtaaaaccgccacaaaccataattaatagctgccgagtct
gaaaaaaactgataattgtcgaaaaccaaccgttagtcatcatttatcacgtcccgaacaagtttgaaagctaaagataa
cttccaaggtccaactcacaacatgaagattactcctgcgtcaatcaccttattgagtttttggtgccagcactaggcc
taacgggatacgattgtcagggacattttatttctgtgaatgttatcaaccatgccataaacagtgatattagttcaaaa
agcggtcgtcatagttatcaggaaaatgctggtctgcatcccgagaacaggagtgcgagaatcagttataatgttgatgg
cgatccatgttcgtatattccacccatcgcaggaccgtgaggctagtgatactaatttttctaaaaagactggggtgcct
gggttgaatatggtcaaaataaggaaattttagaagtgtactcttcgagtgggaaatgcgggccgtgttaa

>*Bgh*Efc102
aacggcaaaagcagtgtttaagaagcttgcacctttcgtcctcatcacgcattttctcatactccttcattttcttcatc
tcatccctcgtctgtcgaggaggtgccgttcaagttctacctgctttcgtcaacaaggccacagcggcaaaatgcgactt
gtcgaaactctcacagccgtacccttaatcttctcggtctacgtctctgcggaacatgggtatatctgttcagatgctga
gggaaatgcaaacacaacctataataaagcattcgtagaggaaagcttgtgttccagacagcgaattgtcagcggccaaa
cttgcggcaaacgtcgaggcgaaaaagtatccccaggtctgggtcgactcggaagattatggcttcaacgagactgtttt

actctggaaatatcaaggaaatag*ctctgagtctctag***gtaatacgtagccaaacttacagctctttttggagacgctaa**
actccccgacagcccgtaactaaccatcatatag*atgagcctaatgcctttgtttttaccaacaaattctgtaatatttt*
gggattattacaggtatctagtgaccagtatgcgatatgc

>*Bgh*Efc103
cagcttccacctcattcgtctttcttggagaacttgaacattctttttccacaatctactctttcttgcctttccagag
cttctctcattcgtcttttaatactgctcaggccgaattttcctacttggtgtccactgagaggaaataattaagcctat
*acacagccaataaacccttcaagcagaagcaaagacgtccattctcaca*Atgaagctccaaaccattactcaactaattg
cgttcctgtgcctttctccaacattttctgcggcacaagat**tataaatgt**ggaaatcaaaaaataaaatcgaaggacatt
aaaaagggccttaaaaatttggctacaccaagtttgactttacagaaaacagttatacaaagtctatactttggaaacgg
t**caaacttgctatattgccaatattcctctaagcaattacaacggtgagcggggtaagcaaatttcatag**

>*Bgh*Efc104
agacattcatcccatcgccaatctagctcgagcacgcatcaaccgtccccttgtccccctcgacatcccgaatcatcaaa
*g*atgcgtacagccactctcctcgcattctttggtacatggcttggctctcttgctggtatagtgcgatttgaacctcaa**t**
atctttgtggtaatgacgttcacattccggtatcacacgtcaatagttccttggatcaagcccaagaacaggcaataaac
tgccaacaaggttttaacataggaagtccgggttatctgtat**gatggcaaacccaagaatagggcaaccggtcaactgta**
ttggcgccctatcaatgttccggaaggtttcggattcatcgatagtaagtttctaccaccatgcatgcttttggctttga

>*Bgh*Efc105
aagggagaaattgtttcacctgcgagatccaagctatcaatacatccagttactacccccaaatataatcaacagttgtc
*accctcaacataattaac*atgaaaagctcagctctcgttttctttgcagctctgttgagctatgcgatgcctgtgcttgg
tgctatacat**tataactgt**ttagatgcctatattcccaaaacagaaatagatgaaagagttcgagcaaaataccaagatt
tggtagctcaaggtacacggcccgaccaattccaagctgggagtacctttggtcatgtgagctataacagggaatctgta
ctaaatcgac**gtatgaatgaatctttccgaaaacatagcattccccaattatactcactggcaatag**atggctcaatgga
aatcacggtaaaatttgataaaagtaaaaatatcatttccgtaaaagctctttactcgggcaataattataactgtcaag
tatttgattgggaatag*gagtctaa*

>*Bgh*Efc106
atctaaaagctgacaatcatcaccttcaccaaagagaaagcacgcaccattgcagaagccttcgtctcttacatcaagga
*cccttctatctgtaaccaag*atgaatctgctctatttcactgaaattgcgacaattctgagtttgtttacattattatgt
accgctaccggtctagatgaaggttatgagatg**tataattgt**tatggtcaaacattttactctaataatgtttataatac
tgctttaaaatcatttaaaatgtcaattggtgaatctgaaggctaccctagaaactatgaagcacctggcttagatggta
ctcctccgtataccatattcccaatcttaaatgaagttctgtttaaaatagctt**gtgagttaaaatattcatacaaattg**
acacctttagttaaagctttatctagctcccaagccatattatttcttagttgtggacggagagggcgctcaagctggta
tggtatatgaaacttcccatggttacgtagcgtgttag*gccaggtgaatggctttaatttctcgca*

>*Bgh*Efc107
agctccagatctctcaaaaatatcaagctcattgtctgagaatctttattgatccgagtcaaaaattattactgctcagc
*tactttttcgaa*atgaggcttctaccactggcttcattcattgcattaatgagccatctgatttcggtgtctgccgaata
caag**tatgtatgt**ccaagcaaaattaaatttacgacagccagaatcgacatgttacggaacgatatatcctctggtagta
ttacagaaggtgtcttgattatcacctcttcaggtctaagagtcttaacctacatctttgacagaaagattattgatggc
g**gtcagtcaatctgttccatttttatcgaaaatagctactgatatttcacgttctaaaag**tcgcgtacgactttatggta
actgtaaattatagggataaattgacaagggtatatgagaaagaaagtgaacatacaagaatttgtgaattgatatttgt
gctggattaa*ccgccagtgtagagacatggtgt*
