# Supplementary material for: Powdery mildew fungal effector candidates share N-terminal Y/F/WxC-motif
Source: BMC Genomics. 2010 May 20;11:317. doi: 10.1186/1471-2164-11-317 (PMC2886064; doi:10.1186/1471-2164-11-317)
Supplement: Additional file 7 — Table S4. Sequences of primers used for semi-quantitative RT-PCR and qPCR. [file 1471-2164-11-317-S7.PDF]

Table S4. Sequences of primers used for semi-quantitative RT-PCR and qPCR.

Gene	Forward Primer (5' to 3')	Reverse Primer (5' to 3')
**Semi-quantitative RT-PCR primers**		
BghEfc1	CCAACTCGAAGTATAGATCCAAAAT	GTGAACTTCCTTCCGCTGG
BghEfc2	ACACCCATAAATTCAATTCGT	AAGCTCAAACAAGATGGAACTA
BghEfc3	CACTCCAAACGCAAACATCACA	TAGCCTGGGTTGTCGAGG
BghEfc4	AGCTGCTACTTCCTATCTGCA	TTAGATACACTCTTCGGGCGA
BghEfc5	TCCGTGAGACGAAATTCTCG	GGAAGGGACATAGTCGCCA
BghEfc6	CCACACATTTTAAATAGTCGACC	AGCCATTTACAACGTCGCATG
BghEfc7	CGCGTCGTTATTCAGCTGTC	CATCTTTATTCTTGTTCGCCTT
BghEfc8	GCCTATTTTCCTTGGCCCTA	CTCGGCGACTTCCTCTTCA
BghEfc9	AAGTGGTTCATAGCGTTCATGT	TTCCTAAACGCGATCTGTCTAA
BghEfc10	GTTGAGGCTGGATATTTGTGTC	CCTATAACCCCTGCCGGT
BghEfc12	CACCATGTATTGGGATTGTGATGATTATAG	CCAGCCTAGTCCTGAAC
BghEfc15	CACCATGTTGGATGGTTACCGATGTG	TGCTATGCATGCCATTTAGC
BghEfc16	CACCATGTCATACTATGACTGCAACGG	CTCAATACATATATTTGCATTCG
BghEfc18	CACCATGATGATAGGCTATCAATGTGAC	CCGTATCATTAATTAAATGGCAC
BghEfc19	CACCATGGAACAATATTTTAAATGTCATTC	GTTAATTCCGGTGGAAAG
BghEfc21	CACCATGGCGGATTATCGTTGTGATAAT	CAGGTTTATTGACAGCTAATTAAG
BghEfc26	CACCATGCGAACGTGGCAATGCCG	CTGCCTAGGTGCATTCTTC
BghEfc27	ATCATTAAGTACGGCTGACGA	AATGTCCTTACACGGTTCGT
BghEfc29	CACCATGGTGCAGTGTTTTGGTGAC	GCCGACTAATACGGGC
BghEfc30	CACCATGTTAATGACTTATGAATGTGTGAG	GTGTTATGAGGCCAGCC
BghEfc35	CACCATGGCCTTCAAGGGGTATCATA	TGTTACCGAACAATGCCAC
BghEfc36	CACCATGCTGGATGGTTACAATTGTCT	CGCATATCATTCAATAATTTC
BghEfc37	GCGCCTGGAGAGTATGTTTA	TAAGGATAGGGCACGGCA
BghHistone H3	CGACTTGCGGTTCCAGTCA	ACACAAATTGGTATCCTCGAAGAGT
Bghβ-tubulin	CAGCAGATGTTTGATCCGAA	AGAACATAGGGCAGTTTGA
**qPCR primers**		
BghEfc1	GCTTTTCTTAAGCCATTTGA	TTCGTTTCAACCTCTTCTTC
BghEfc2	CATCTTGTTTGAGCTTGTCA	CCATACTACCCCAAATAGCA
BghEfc3	ACAGAGCTGTCGCTAGATTC	TTTCTATTGGCTGTTCCTA
Bghβ-tubulin	ATCGTATCCGCTACTTCAGA	CTGAGGCCGAGTCTAATATG
HvGAPDH	GTGAGGCTGGTGCTGATTACG	TGGTGCAGCTAGCATTTGAGAC
